# A universal method for high-quality RNA extraction from plant tissues rich in starch, proteins and fiber

**DOI:** 10.1038/s41598-020-73958-5

**Published:** 2020-10-09

**Authors:** Amaranatha R. Vennapusa, Impa M. Somayanda, Colleen J. Doherty, S. V. Krishna Jagadish

**Affiliations:** 1 Department of Agronomy, 2004 Throckmorton Plant Sciences Center, Kansas State University, 1712 Claflin Road, Manhattan, KS 66506-5501 USA; 2 Department of Molecular and Structural Biochemistry, North Carolina State University, Raleigh, NC 27695 USA

**Keywords:** Plant sciences, Plant stress responses, Abiotic

## Abstract

Using existing protocols, RNA extracted from seeds rich in starch often results in poor quality RNA, making it inappropriate for downstream applications. Though some methods are proposed for extracting RNA from plant tissue rich in starch and other polysaccharides, they invariably yield less and poor quality RNA. In order to obtain high yield and quality RNA from seeds and other plant tissues including roots a modified SDS-LiCl method was compared with existing methods, including TRIZOL kit (Invitrogen), Plant RNeasy mini kit (Qiagen), Furtado (2014) method, and CTAB-LiCl method. Modifications in the extraction buffer and solutions used for RNA precipitation resulted in a robust method for extracting RNA in seeds and roots, where extracting quality RNA is challenging. The modified SDS-LiCl method revealed intense RNA bands through gel electrophoresis and a nanodrop spectrophotometer detected ratios of ≥ 2 and 1.8 for A260/A230 and A260/A280, respectively. The absence of starch co-precipitation during RNA extraction resulted in enhanced yield and quality of RNA with RIN values of 7–9, quantified using a bioanalyzer. The high-quality RNA obtained was demonstrated to be suitable for downstream applications, such as cDNA synthesis, gene amplification, and RT-qPCR. The method was also effective in extracting RNA from seeds of other cereals including field-grown sorghum and corn. The modified SDS-LiCl method is a robust and highly reproducible RNA extraction method for plant tissues rich in starch and other secondary metabolites. The modified SDS-LiCl method successfully extracted high yield and quality RNA from mature, developing, and germinated seeds, leaves, and roots exposed to different abiotic stresses.

## Introduction

Modern biotechnological advances in functional genomics and the access to whole-genome sequences are invaluable tools for crop improvement. Identifying the molecular factors affecting crop growth and productivity under harsh environmental conditions and evaluating their functional responses will help decode pathways enhancing stress-tolerance in crops. Deciphering these molecular factors and stress tolerance mechanisms demands high-quality RNA, which is a pre-requisite for various techniques in molecular biology, including gene expression quantification by RT-PCR, RT-qPCR, RNA-seq, northern blot analysis, construction of cDNA libraries and PCR amplification-based gene cloning.

Seeds are complex storage organs, composed of embryo and endosperm covered by a hard seed coat. The endosperm, which occupies the major proportion of the seed, contains significantly high quantity of starch followed by other polysaccharides (cellulose [dietary fiber], arabinoxylan, β-glucan and fructans), proteins, lipids, and secondary metabolites. Most of these components in seeds have structural similarities with nucleic acids and hence co-precipitate along with RNA, thus challenging the extraction of high yield and quality RNA^1–5^. Several RNA isolation methods have been reported to yield quality RNA from wheat and other seeds rich in starch, polysaccharides, and other secondary metabolites^1,2,6^. A few limitations of existing RNA extraction methods mentioned above include—(i) need more than one extraction buffers^1,2^, (ii) methods developed are restricted to extracting RNA only from seeds, (iii) require expensive TRIZOL and/or commercial kits for extraction^6^ and comparatively cumbersome^1,2^. Hence, there is a need to develop a rapid and universal RNA extraction method using basic laboratory chemicals that can be applicable to a wide range of tissues containing interfering substances that alter the quality of extracted RNA.

RNA isolation methods with guanidine isothiocyanate based extraction buffers fail to extract quality RNA from seeds rich in starch, as guanidine isothiocyanate induces solidification of starch^1^. Solidification of starch enhances the co-precipitation of starch/polysaccharides along with RNA due to their structural similarities, for example presence of ribose sugar. The large polysaccharide fractions in the seed can physically trap RNA and be entrained during centrifugation and gets discarded during the phase separation, thus leading to low yield. Additionally, small polysaccharide particles can be partitioned into the aqueous phase during phase separation and co-precipitate along with RNA, which further reduces RNA yield and renders it less suitable for downstream applications^1,7–9^. Therefore, to overcome the starch solidification encountered by existing methods, additional purification steps or alternative methods are required^1,6,10,11^. In addition, extraction of high-quality RNA from plant tissues subjected to different environmental stresses such as heat, drought and cold stresses can be difficult due to increased accumulation of high molecular weight polysaccharides and secondary metabolites that interfere with RNA isolation. Secondary metabolites in the oxidized form irreversibly bind to the nucleic acids and act as inhibitors for downstream applications or degrade the RNA^4,12–15^. Isolating high-quality RNA free from proteins, polyphenols, and polysaccharide contaminants from samples exposed to abiotic stresses is essential for downstream applications. Moreover, the quality and quantity of RNA extracted using existing RNA extraction methods and commercial kits differ depending on the plant species, genotype and tissue type^3,16,17^. Hence, it is evident that a universal RNA extraction method that can be used across different plant parts, including seeds, which contain high levels of starch and secondary metabolites and roots would be of great value to the crop science community.

The major objective of the present study was to develop a robust and reliable RNA extraction method to obtain high-quality RNA from different plant tissues, including roots and seeds exposed to different abiotic stresses. To achieve this objective, five RNA extraction methods, i.e. TRIZOL kit (Invitrogen), Plant RNeasy mini kit (Qiagen), Furtado^6^ method, CTAB-LiCl method^3^, and our modified SDS-LiCl method, were used to compare the quantity and quality of RNA extracted from mature wheat seeds collected from field-grown plants exposed to ambient, and high night temperatures (for details on crop management and stress imposition see Hein et al.^18^). The extracted RNA was then used to assess the quality through RT-qPCR analysis. In addition, RNA from different tissues of wheat plants including mature, developing and germinated seeds, leaf, and roots, exposed to cold, freezing, and high night temperature stresses were isolated using the modified SDS-LiCl method. The robustness of our modified SDS-LiCl method was further tested on developing sorghum seeds and developing and mature maize seeds, obtained from field grown plants. Given the variability of RNA isolation methods, the ability to achieve comparative analysis of genomes and transcriptomes across crops and tissue types continues to be a challenge for researchers. Here we describe a universal method that can be applied in seed tissues containing high starch and other plant tissues including roots, which contain high fiber and secondary metabolites. Our intent in presenting this modified method is not to oppose other methods that work well for specific crops and tissues, but rather to present a universal method that is more robust, rapid and works equally well across different field crops and plant tissues.

## Materials and methods

Two winter wheat (*Triticum aestivum* L.) genotypes Tascosa and Tx86A5606 with contrasting responses to high night temperature (HNT) stress were used to isolate RNA from different plant tissue and growth conditions. RNA was isolated from physiologically mature wheat seeds collected from field grown plants^18^, flag leaves from controlled environment chamber grown plants exposed to HNT and control conditions^19^, germinated seeds under cold stress, roots of wheat seedlings grown on Murashige and Skoog (MS) media exposed to freezing stress. Our modified SDS-LiCl method was extensively compared to other currently available methods developed by public institutes and private companies. RNA was also isolated from plant samples exposed to cold, freezing and HNT stress to demonstrate that the efficiency of the new method can be extended to plants exposed to different abiotic stress conditions. In addition, the method was adopted for extracting RNA from developing and mature seeds of field-grown maize and sorghum. Details of the sample collection and stress imposition and associated references are detailed below.

### Tissue sampling from control and high night temperature (HNT) stress exposed plants

#### Mature seeds

Physiologically mature wheat seeds were collected from field grown plants, 21 days after 50% flowering. The field experiment was carried out at Kansas State University, Agronomy research farm at Manhattan, Kansas. High night temperature (HNT) stress was imposed by placing custom built field-based heat tents on already established wheat plants from 10 days after 50% flowering till maturity^18^. The heat tents maintained temperatures < 1 °C higher compared to ambient conditions during the day. Higher night-time (19:00 h to 6:00 h) temperature was achieved using a custom built semi-automated heating system operating independently in each tent during grain filling (For additional details on the construction, operation, and temperature imposition see Hein et al.^18^). Physiologically mature seeds were collected from two winter wheat genotypes (Tascosa and Tx86A5606) grown under ambient night temperature (control; 22.7 °C) and an elevated night temperature of + 3.2 °C (HNT stress) during the grain filling, frozen in liquid nitrogen, and stored at − 80 °C for RNA extraction.

#### Developing seeds

Developing wheat seeds were collected from controlled environment chamber grown plants at 14 days after heading/HNT stress exposure, from control and HNT treatments. Two winter wheat genotypes, SY Monument (Tolerant) and KS07077M-1 (Sensitive) contrasting for HNT stress were exposed to control (28/15 °C) and a range of HNT treatments (28/18 °C; 28/23 °C; 28/25 °C and 28/27 °C) using the controlled environment chamber facility, at the Department of Agronomy, Kansas State University (For additional details on the high night-time temperature stress imposition see Impa et al.^20^). HNT stress treatments were imposed immediately after the main spike (first emerged) had completed flowering and maintained until physiological maturity. Three biological replicates of main spikes from all the five night temperature treatments were collected at 14 days after the stress was initiated, and immediately frozen in liquid nitrogen, and stored at − 80 °C for RNA extraction.

#### Flag leaves

Flag leaf samples were collected from the controlled environment chamber grown wheat genotypes, Tascosa and Tx86A5606 at 14 days after heading/HNT stress exposure, from control (26/15 °C) and HNT (26/23 °C) treatments. HNT stress treatments were imposed immediately after the main spike (first emerged) had completed flowering and maintained until physiological maturity (For additional details on the HNT stress imposition see Impa et al.^19^). Three replicate samples were collected for each genotype and treatment. Samples were immediately frozen in liquid nitrogen and stored at − 80 °C for RNA extraction.

### Germinated wheat seeds exposed to cold stress

Seeds of the two wheat genotypes (Tascosa and Tx86A5606) were germinated in plastic Petri dishes (100 mm by 20 mm). Seeds were sterilized with 70% ethanol for 2 min, followed by 3% sodium hypochlorite for 5 min. Subsequently, seeds were rinsed 2 to 3 times with sterile distilled water before placing them on the germination paper. Ten seeds were placed in each petri dish containing the germination paper moistened with sterile distilled water. The Petri dishes were incubated in an incufridge (RevSci, Minnesota, USA) maintained at 15 °C to impose cold stress, and at 30 °C for control treatment^21^. The germinated seeds were collected five days after germination was initiated and immediately frozen in liquid nitrogen and stored at − 80 °C for RNA extraction. The sample collection was timed to ensure that each germinating seed had five days of continued cold stress after initiation of germination.

### Wheat roots exposed to freezing stress

Seeds of Tascosa and Tx86A5606 were surface sterilized with 70% ethanol for 2 min and rinsed twice with sterile distilled water. Subsequently, the seeds were washed with 3% sodium hypochlorite for 5 min and rinsed three times with sterile distilled water. Later, the seeds were dried under laminar airflow and were inoculated into sterile test tubes containing half-strength Murashige and Skoog (MS) media (Phytotechnology laboratories, KS) with 0.6% w/v solidified agar. The test tubes were incubated in a growth chamber maintained at 25 °C with 16/8 h day/night photoperiod. After ten days of incubation, the seedlings were exposed to a freezing temperature of − 4 °C in the freezer for 16 h. At the end of the 16 h stress exposure, root samples from control and freezing temperatures were collected into liquid nitrogen and immediately stored at − 80 °C for RNA extraction.

### Sorghum and corn seed samples

The developing seeds of Sorghum hybrid (Pioneer 87P06) and developing and mature seeds of Corn (Pioneer P0805AM) hybrid were collected from the plants grown under field conditions at Kansas State University, Agronomy research farm. The samples harvested in the field were immediately frozen in liquid nitrogen and stored at − 80 °C for RNA extraction.

### RNA extractions

All the solutions used in the RNA extraction procedure were prepared with autoclaved 0.1% DEPC (diethyl pyrocarbonate) treated MilliQ water. All the plastic- and glassware and mortar and pestle were pretreated with DEPC and autoclaved.

### Methods followed to extract total RNA

*Ambion TRIZOL reagent Kit* (by Life Technologies Invitrogen, CA, USA): Total RNA was extracted according to the manufacturer's instructions. Extraction buffer used in this method contains guanidium thiocyanate and RNA was precipitated in isopropanol.

*RNeasy Plant Mini Kit* (Qiagen, Valencia, CA, USA): RNA was extracted according to the manufacturer's instructions. The method uses guanidium thiocyanate based lysis buffer and RNA purification was done using silica-membrane column.

Furtado^6^* method:* The total RNA was isolated based on the combination of both TRIZOL and RNeasy Plant Mini Kit (Qiagen) method. RNA extraction was carried out in the TRIZOL reagent, and the collected aqueous phase was extracted in RLC buffer, followed by steps in RNeasy Plant Mini Kit manufactures method.

*CTAB-LiCl method:* RNA was extracted based on the method described by White et al.^3^. In this method, ionic detergent cetyltrimethylammonium bromide (CTAB) was used in the extraction buffer, and LiCl was used to precipitate RNA.

**Figure 1:**
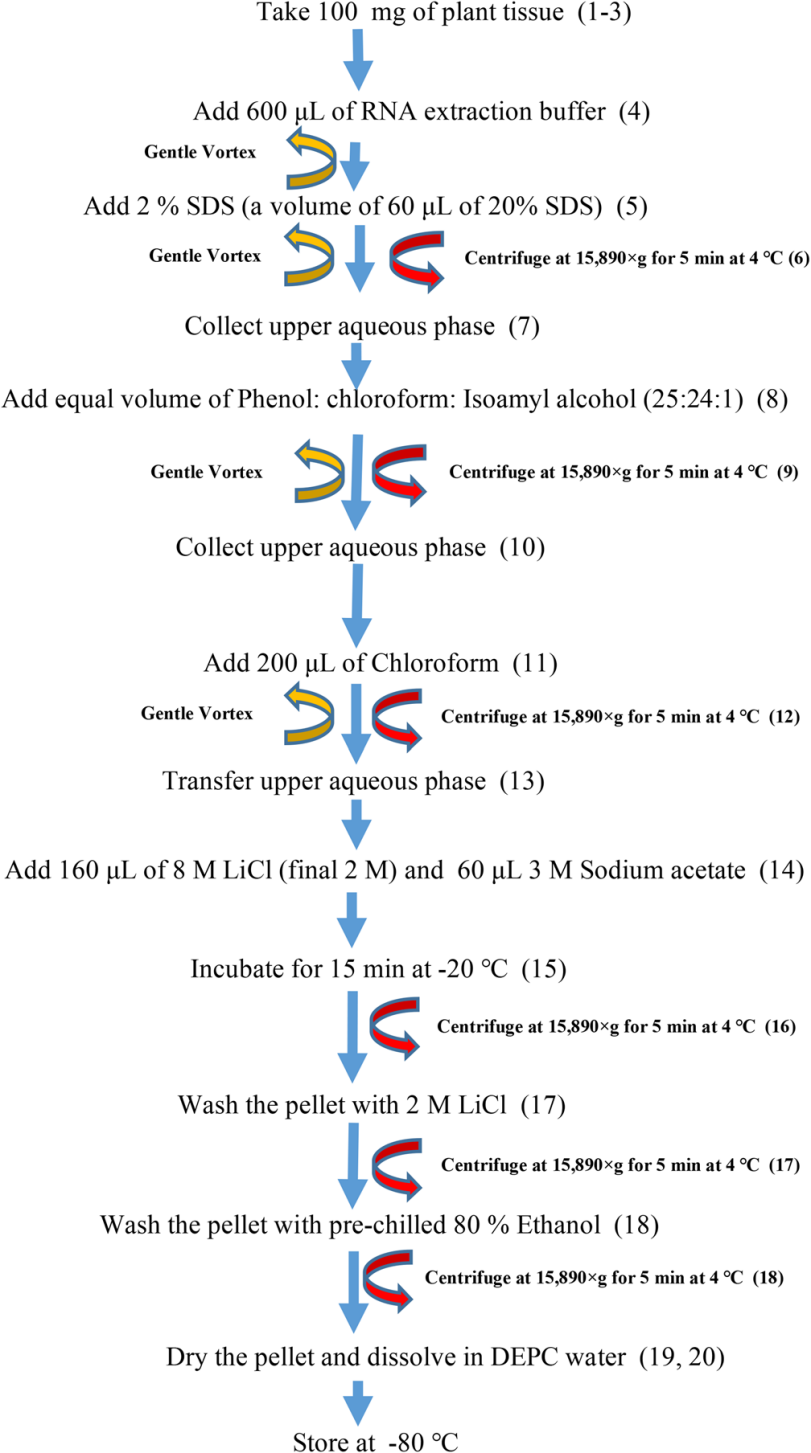
Flow chart describing the major steps involved in the total RNA extraction using the modified SDS-LiCl method. Information for each step (numbers in parenthesis) is detailed in the procedure section.

*New Modified method (SDS-LiCl):* This method follows the SDS-Phenol based extraction and RNA precipitation with sodium acetate and LiCl (Fig. 1).

### Materials and equipments’ and their set up, step by step procedure and troubleshooting are provided in supplementary document

#### Qualitative and quantitaive analyses of RNA

The quantity and quality of RNA were assessed by measuring the optical density (OD) at 260 nm and 280 nm by a Nanodrop-2000 Ultraviolet Spectrophotometer (Thermo Fisher, MA, USA). The RNA quality of the sample was measured by monitoring the absorbance at A260/280 and A260/230 ratios (Table 1). The purity and integrity of the total RNA bands were evaluated by loading 200 ng of RNA samples on 1% agarose gel stained with ethidium bromide and pictured under UV light in GEL DOC XR + and CHEMIDOC XRS + imaging Systems containing the IMAGE LAB software (Biorad, CA, USA). Further, the total RNA was analyzed with the Total RNA 6000 Nano Kit (Vers. II), precisely optimized for total RNA analysis with Agilent 2100 Bioanalyzer with RNA 6000 Nano Lab Chip (Agilent Technologies, CA, USA). The RIN (RNA integrity number) values were calculated using the Eukaryote Total RNA Nano Assay^2^. The electropherograms were analyzed using Agilent 2100 Expert B.02.06 SI648 software composed of data collection, presentation, and interpretation functions.

**Table 1:** Comparative evaluation of A260/A280 and A260/A230 ratios of RNA extracted from mature wheat seeds using different RNA isolation methods, using Nanodrop ND-1000UV spectrophotometer for assessing the RNA quality.

Sample	Ambion TRIZOL	RNeasy plant mini kit	Furtado^6^	CTAB-LiCl method	Modified SDS-LiCl method
**A260/A280 ratio**					
Tx control	1.70 ± 0.02	3.14 ± 2.70	1.68 ± 0.06	2.00 ± 0.09	2.22 ± 0.08
Tx HNT stress	1.76 ± 0.05	4.96 ± 6.10	1.86 ± 0.18	2.10 ± 0.06	2.15 ± 0.09
Tascosa control	1.82 ± 0.10	3.60 ± 3.70	1.64 ± 0.18	2.14 ± 0.06	2.20 ± 0.01
Tascosa HNT stress	1.88 ± 0.08	3.20 ± 2.46	1.71 ± 0.10	2.08 ± 0.07	2.18 ± 0.02
**A260/A230 ratio**					
Tx control	0.39 ± 0.20	0.02 ± 0.03	0.17 ± 0.11	2.09 ± 0.22	2.32 ± 0.02
Tx HNT stress	0.97 ± 0.82	0.02 ± 0.02	0.38 ± 0.02	1.88 ± 0.48	2.26 ± 0.05
Tascosa control	0.51 ± 0.14	0.03 ± 0.02	0.63 ± 0.15	1.98 ± 0.25	2.33 ± 0.06
Tascosa HNT stress	0.83 ± 0.28	0.03 ± 0.02	0.69 ± 0.27	2.19 ± 0.08	2.31 ± 0.08

Control samples were collected from plants grown under ambient temperature, while high night temperature (HNT) stress samples were collected from plants grown under custom built heat tents with + 3.2 °C higher night temperature than ambient temperature (22.7 °C) during grain filling (Hein et al.^18^). Values presented are the mean ± standard deviation of three technical replicate samples. Tx-Tx86A5606.

### cDNA synthesis, PCR gene amplification and RT-qPCR

The RNA extracted from developing and mature seed samples were subjected to DNase 1 treatment (Thermo Scientific, Waltham, MA, USA) to eliminate remaining traces of genomic DNA (gDNA). The first strand of cDNA was synthesized using RevertAid First Strand cDNA Synthesis Kit (Thermo Scientific) according to the manufacturer’s instructions. For polymerase chain reaction (PCR) amplification of 5-enolpyruvyl-shikimate-3-phosphate synthase (EPSPS) gene product (1229 bp), ~ 100 ng of cDNA samples of total RNA extracted from the mature wheat seed tissue using five different RNA extraction protocols were added to a 25 µl PCR reaction mix containing GoTaq G2 Green Master Mix (Promega, Madison, WI, USA) and 0.4 µM of each gene-specific primer (EPSPSF- AAGTCGCTMTCCAAYCGRATCCT and EPSPSR- GGGAAGGTCTTNCGGGTGCA). The amplification was carried out in a thermal cycler (T100 Thermal Cycler, Bio-Rad Inc, Hercules, CA, USA) with PCR conditions as follows: 5 min denaturing at 95 °C; 32 cycles of 30 s denaturation at 95 °C, 30 s annealing at 55 °C, 45 s elongation at 72 °C and a final extension for 10 min at 72 °C.

For RT-qPCR analysis, the following reference and gene-specific primers were used to measure the corresponding genes (Fwd/Rev): *Actin* (Forward- GCCATGTACGTCGCAATTCA, Reverse-AGTCGAGAACGATACCAGTAGTACGA) and *Beta-amylase* (*BMY*) (Forward-CTAGCCAACTATGTCCAAGTCTACGT, Reverse- ACTGTGGGATGGGGATGTTGACGA). Quantitative PCR/Real-Time PCR (RT-qPCR) was performed to determine the quality of the RNA extracted from the seed samples using the *BMY* (Beta Amylase) gene. For all the RT-qPCR analysis three technical replicates were used for each of the three biological replicates and RT-qPCR was performed with a reaction mixture containing 8 μl of SYBR Green master mixture (Applied Biosystems SYBR Green, Carlsbad, CA, USA), 2 μl each of forward and reverse primers (5 μM), and 20 ng cDNA (2 μl) to make the final reaction volume of 14 μl. RT-qPCR (CFX96-Touch Real-Time PCR Detection System, Bio-Rad Inc, Hercules, CA, USA) was performed at 95 °C for 5 min, and 40 cycles of 95 °C for 30 s and 58 °C for 30 s. Relative gene expression was quantified using the 2^−ΔΔCT^ method as described in Livak and Schmittgen^22^, and *BMY* gene expression was normalized using *Actin* as a reference gene. A melt curve profile was incorporated in the thermal cycling method to check the specificity (with no gDNA contamination, no primer dimers, and no non-specific product) of the RT-qPCR reaction.

## Results and discussion

### SDS-LiCl method extracted higher yield and quality RNA from seeds

**Figure 2:**
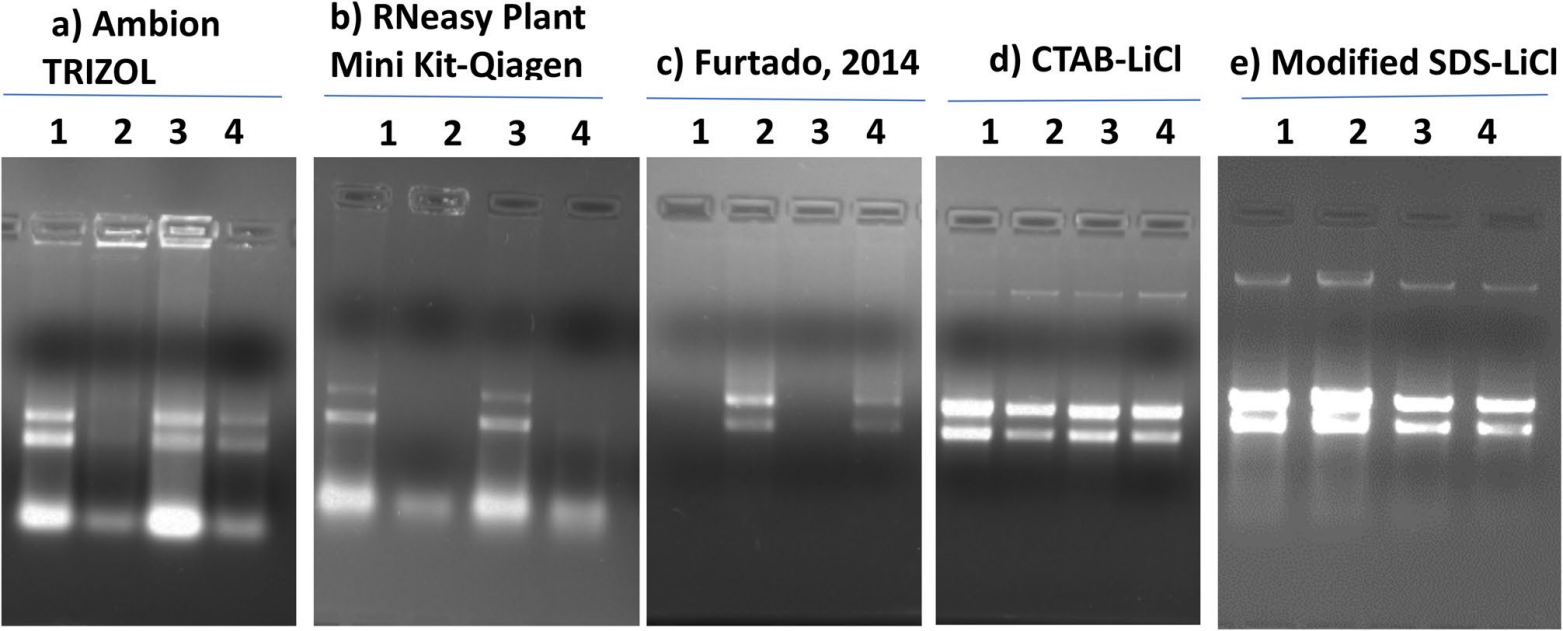
Agarose gel electrophoresis of mature seed RNA extracted using 5 different RNA extraction methods. Total RNA was extracted from mature wheat seeds collected from plants grown under ambient and post-flowering high night temperature (HNT) stress conditions in field (Hein et al.^18^). The five extraction methods are Ambion TRIZOL (**a**), RNeasy Plant Mini Kit (Qiagen) (**b**), Furtado, 2014^6^ method (**c**), CTAB-LiCl method (**d**), and modified SDS-LiCl method (**e**). The total RNA from wheat seeds of four different genotypes are numerically labeled from 1 to 4. 1: Tx Control, 2: Tx HNT stress, 3: Tascosa Control and 4: Tascosa HNT stress. Tx- Tx86A5606. The agarose gel electrophoresis images used in this figure for the selected genotypes were cropped from the original source gel images provided in the supplementary source Figs. 1 and 2. The cropped gel images selected from the source gel images documented at high and low contrasts were used to obtain the visible RNA bands (low and high intensity) in the gel images and the cropped images were auto corrected for the same brightness/contrasts using PowerPoint image tools.

**Figure 3:**
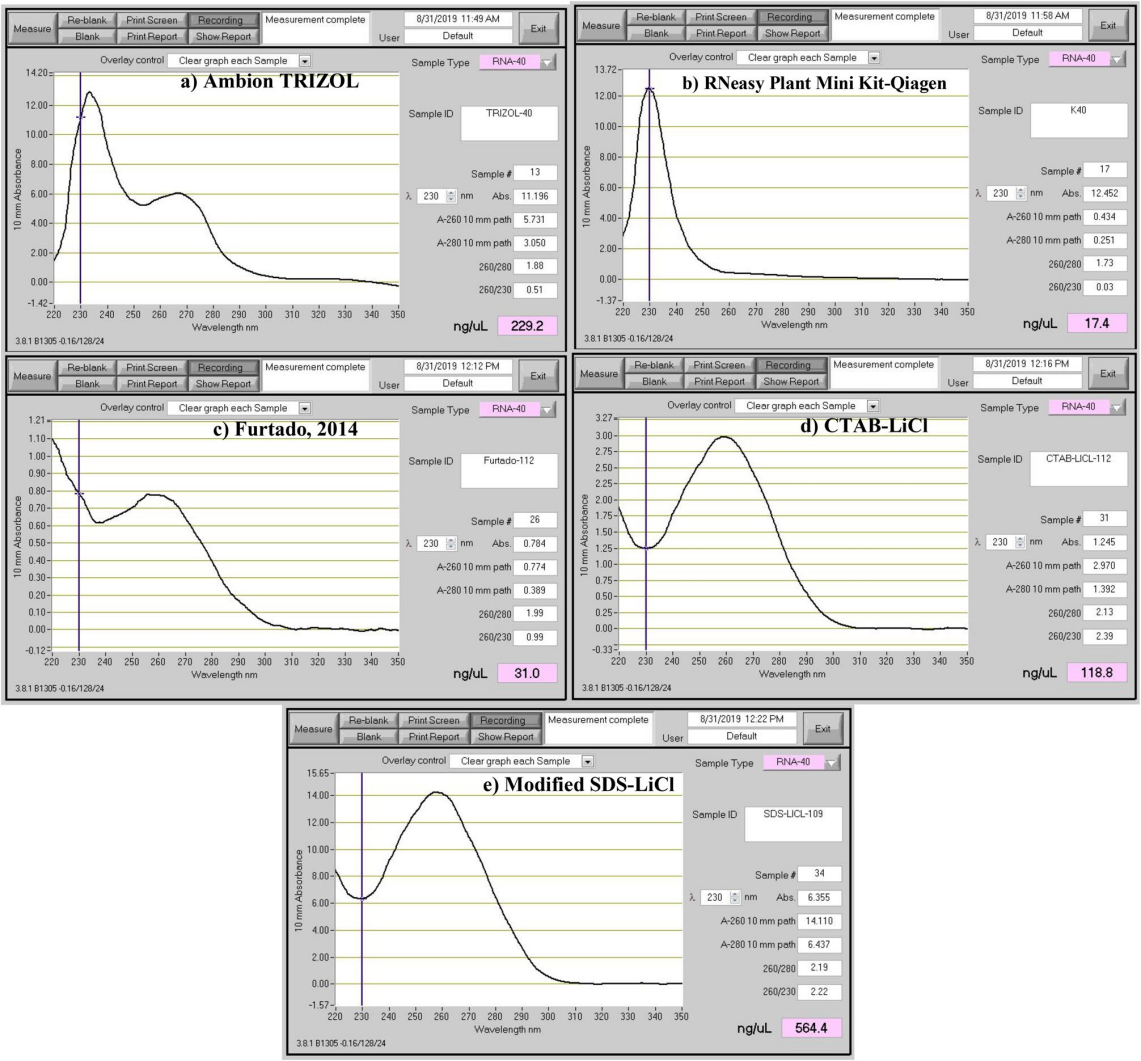
Absorbance spectrum of the RNA samples isolated from mature wheat seeds using different extraction methods. The RNA extraction methods are, Ambion TRIZOL (**a**), RNeasy Plant Mini Kit (Qiagen) (**b**), Method of Furtado^6^ (**c**) CTAB-LiCl method (**d**), and modified SDS-LiCl method (**e**). The absorbance peaks at 220 (organic contaminants), 260 (nucleic acids) and 280 nm (protein) shows the quality and impurities of RNA samples extracted using 5 different methods.

Previous methods using guanidine isothiocyanate based RNA extraction buffer results in starch solidification and co-precipitation of RNA with starch, reducing the quantity and quality of RNA^1, 2,23,24^. Modified SDS-LiCl method described here provides a robust method to obtain high-yield and high-quality RNA from wheat seeds containing high starch and other polysaccharides, and other tissues such as roots that are rich in fiber. In addition, this method has the potential to extract high yield and quality RNA from developing and mature seeds exposed to different abiotic stresses. Analysis of total RNA from mature wheat seed on agarose gel electrophoresis revealed that ribosomal RNA bands were intact and intense, demonstrating that the total RNA extracted by modified SDS-LiCl was not degraded (Fig. 2e). However, other methods produced faint bands of RNA, with higher impurities in the gel-well and a smeary background during the run (Fig. 2a–d). Absorbance spectra of RNA with a broad peak pattern at 260 nm (Fig. 3) and ratios of A260/A280 and A260/A230 being close to or above 2 (Table 1) for all the RNA samples extracted using modified SDS-LiCl method. This demonstrates the RNA extracted to be free from protein contaminants, organic solvents, and other secondary products compared to other RNA isolation methods.

**Figure 4:**
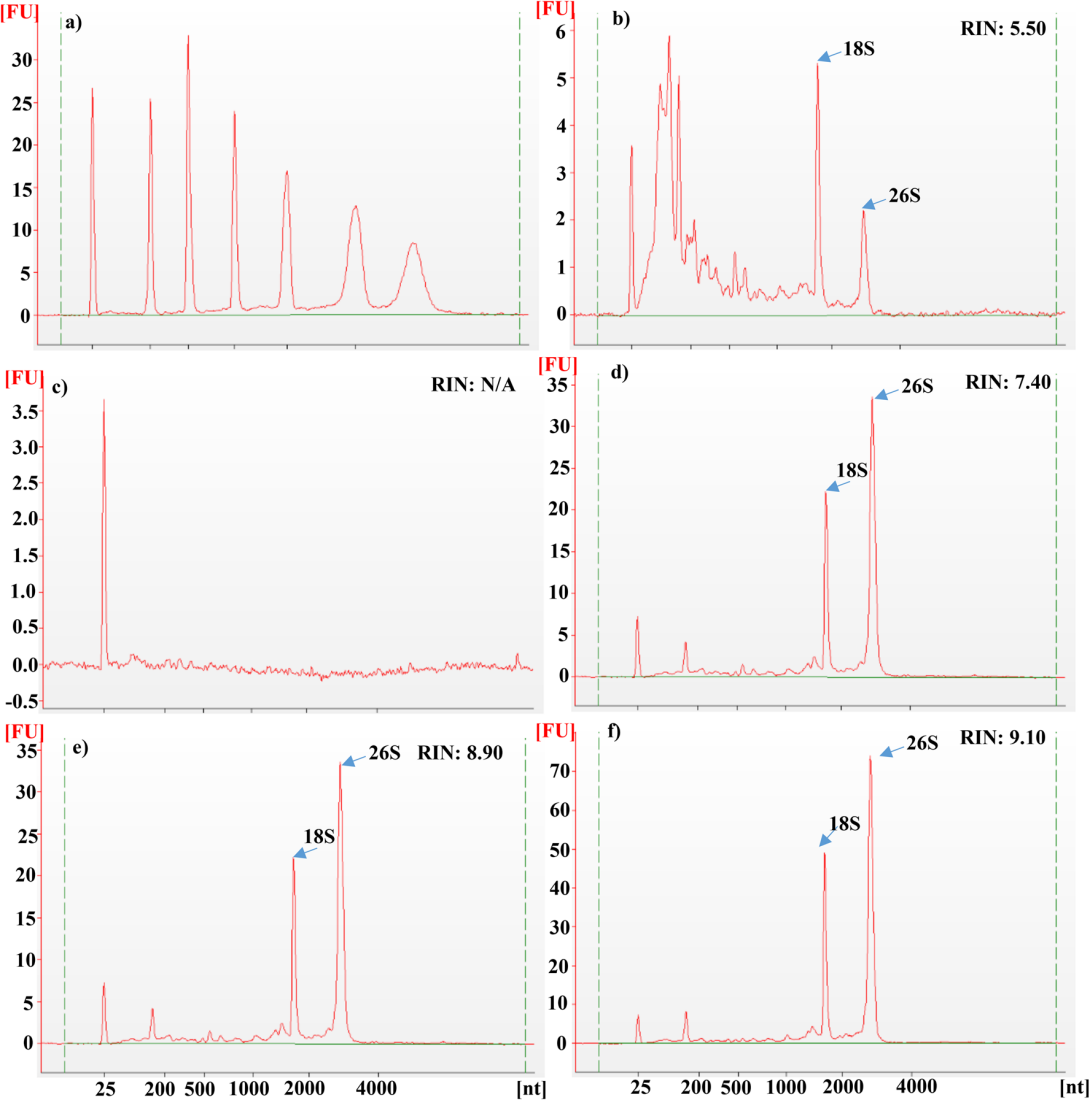
Electropherograms of total RNA of mature wheat seeds extracted using different RNA isolation methods with the Eukaryote Total RNA Nano Assay on the Agilent 2100 Bioanalyzer. X-axis units in nt (Nucleotides); Y-axis units in FU (Fluorescence Units). Electropherograms of RNA ladder (**a**), Ambion TRIZOL (**b**), RNeasy Plant Mini Kit (Qiagen) (**c**), Furtado^6^ method (**d**), CTAB-LiCl method (**e**), and modified SDS-LiCl method (**f**). RIN: RNA integrity number.

Evaluation of total RNA quality using Agilent bioanalyzer 2100 revealed superior quality of RNA extracted using modified SDS-LiCl method with the electropherograms showing distinct, prominent peaks for ribosomal RNAs (rRNAs), whereas other methods exhibited either many small peaks and/or no protuberant peaks for ribosomal RNAs (Fig. 4 and Supplementary Figs. S1–S5). RIN values determined on Bioanalyzer (Agilent 2100) using Expert software for total RNA extracted using modified SDS-LiCl method ranged from 7.6 to 9.1 (Table 2). The capillary gel electrophoresis virtual gel images (Agilent 2100 Expert B.02.06 SI648) revealed the intact rRNA bands of high quality RNA isolated by modified SDS-LiCl method compared to RNA extracted using other methods, except CTAB-LiCl method, which was equally effective in extracting high quality RNA (Supplementary Figs. S1–S5). Total RNA isolated with modified SDS-LiCl method produced significantly higher RNA yield (ranging from 19.41 to 37.24 μg/100 mg FW) compared to other methods including CTAB-LiCl method, which was on average 80% lower compared to modified SDS-LiCl method (Table 2). Similarly, lower RNA yield from wheat seeds using CTAB-LiCl has been reported^23^. The CTAB, as a cationic surfactant, favorably interacts with the anionic nucleic acids (DNA and RNA), including proteins and other biopolymers (polysaccharides and secondary metabolites). Therefore, due to the higher affinity of CTAB with both nucleic acids and other biopolymers, leads to reduced yield of RNA. On the other hand, anionic surfactant SDS does not interact with nucleic acids (anionic) but highly reactive with proteins and other polysaccharides and forms aggregates. Thus, SDS is more effective in separating RNA from other biopolymers during RNA extraction^25–29^. Moreover, CTAB-LiCl method has a lengthy procedure and is time consuming compared to the modified SDS-LiCl method. Higher RNA yield with the modified SDS-LiCl extraction method suggests the absence of starch interference during RNA precipitation.

**Table 2:** Comparative quantification of mature wheat seed RNA yield evaluated by Nanodrop ND-1000UV spectrophotometer and RNA integrity numbers analyzed by Bioanalyzer (Agilent 2100) for assessing the quantity and quality of RNA extracted using different isolation methods.

Sample	Ambion TRIZOL	RNeasy plant mini kit	Furtado^6^ method	CTAB-LiCl method	Modified SDS-LiCl method
**RNA Yield** (**μg/100 mg FW**)					
Tx Control	21.12 ± 13.54	0.55 ± 0.23	0.84 ± 0.10	6.74 ± 1.17	28.99 ± 2.12
Tx HNT stress	23.09 ± 15.58	0.44 ± 0.17	3.95 ± 0.17	4.94 ± 1.05	19.41 ± 1.23
Tascosa Control	28.22 ± 21.53	0.62 ± 0.39	1.51 ± 0.91	6.09 ± 1.73	31.22 ± 1.24
Tascosa HNT stress	17.04 ± 07.04	0.56 ± 0.24	1.24 ± 0.81	5.46 ± 1.40	37.24 ± 1.29
**RIN** (**RNA integrity number**) **values**					
Tx Control	5.50	NA	NA	9.00	7.60
Tx HNT stress	2.60	NA	8.10	9.30	8.40
Tascosa Control	6.50	NA	NA	8.90	9.10
Tascosa HNT stress	2.70	NA	7.30	9.10	8.80

Control samples were collected from plants grown under ambient temperature, while high night temperature (HNT) stress samples were collected from plants grown under custom built heat tents with + 3.2 °C higher night temperature than ambient night temperature (22.7 °C) during grain filling (Hein et al.^18^). Tx-Tx86A5606. Values presented are mean ± standard deviation of three technical replicate samples. NA – Not available; these samples had low quality RNA and hence RIN values were not obtained from the bioanalyzer.

### Components that make SDS-LiCl effective in extracting quality RNA from seeds and other tissue

Even though the extraction buffer used in the SDS-LiCl method contains NaCl, PVP, Tris/HCl, EDTA, β-mercaptoethanol, and SDS (see supplementary document), similar to existing methods, we optimized the concentrations of these chemicals for seeds and other tissues including roots. The chemical composition confirmed that 2.5 M NaCl, 2.5% PVP, 100 mM Tris, 25 mM EDTA, 2.5% β-mercaptoethanol, and 2% SDS, helps obtain high quality and yield of RNA without the interference of starch and other impurities. The enhanced yield and quality of RNA achieved in the modified SDS-LiCl method compared to CTAB-LiCl is due to the addition of SDS, and higher concentration of other chemicals (PVP and NaCl) in the extraction buffer. In agreement with previous studies, an increased concentration of NaCl, PVP, β- mercaptoethanol and SDS in the extraction buffer of the modified SDS-LiCl method, not only promotes cell lysis but also prevents RNAse and polyphenols in the isolated RNA^1,2,5,8,30–32^. In addition, post-grinding addition of SDS directly to the homogenate suppressed excessive foaming and facilitated proper lysis of seed samples and prevented starch solidification^1,2, 33^. Additionally, in the modified SDS-LiCl method, phase purification using acidic phenol (pH 4.3) saturated with citrate buffer helped to improve the RNA quality by separating RNA from bulk starch residues compared to CTAB-LiCl method. Efficient separation of bulk starch residues and degraded proteins during the centrifugation of RNA containing aqueous phase using acid phenol saturated with 0.1 M citrate buffer (pH 4.3) supports earlier findings^2^.

A combination of 2 M Lithium chloride and 1/10 volumes of 3 M sodium acetate (pH 4.8) possibly facilitated better RNA precipitation. This is in agreement with previous studies wherein lithium chloride and sodium acetate were used to promote precipitation and resulted in high RNA yield^2,3,34^. Adding both lithium chloride and sodium acetate in the precipitation step enhanced the yield, whereas in CTAB-LiCl method, there was no phenol separation and LiCl alone was used for precipitation. Overall, the RNA obtained from modified SDS-LiCl method from field-grown wheat seeds under both control and HNT stress had high yield and quality, demonstrating a marked improvement compared to all other existing methods.

### High quality RNA extracted using SDS-LiCl method suitable for downstream applications

PCR amplification of EPSPS gene full length CDS with 1.2 kb product size and a prominent band provides support that cDNA obtained from RNA using the modified SDS-LiCl method is high quality compared to other methods. In addition, it indicates that RNA integrity is sufficient to amplify large size gene fragments (Supplementary Fig. S6). The RT-qPCR results of total RNA quantification show a tendency to obtain lower Cq values for the cDNA from RNA samples extracted by the modified SDS-LiCl method compared to other four different methods (Supplementary Table S1). When comparing the reproducibility expressed in terms of SEM values, the best results were obtained for samples extracted by the modified SDS-LiCl method. Comparatively, using other methods, high Cq values and SEM were obtained suggesting that RNA extracted using modified SDS-LiCl method resulted in high quality cDNA with lesser interference of contaminants.

**Figure 5:**
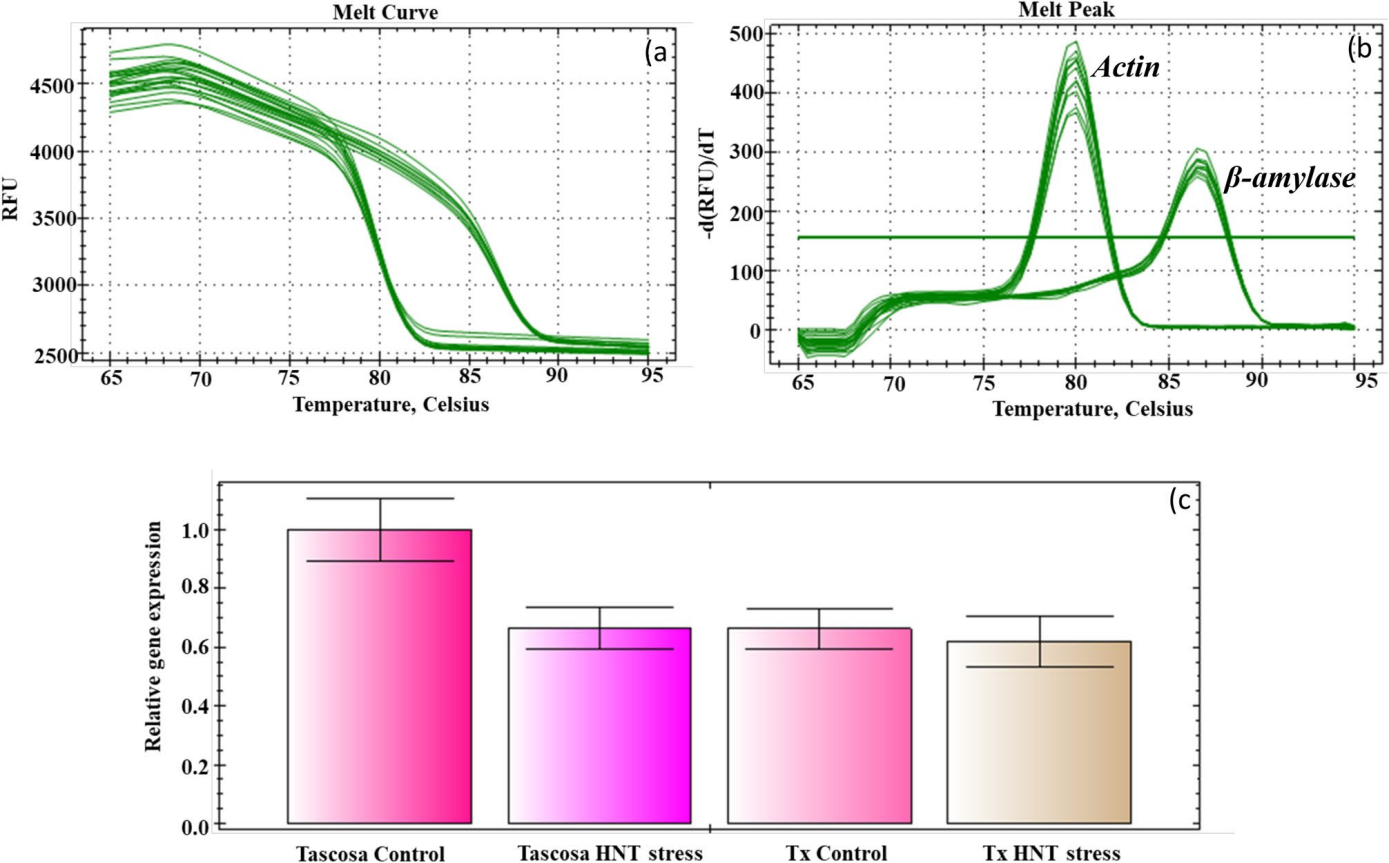
Quantitative real-time PCR (RT-qPCR) assay using the RNA extracted from modified SDS-LiCl method. (**a**) Amplification curves shows the Ct values of PCR amplification fragments of two genes (*Actin* and *β-amylase*) from cDNA synthesized using RNA extracted from the modified SDS-LiCl method and no reverse transcriptase (RT) controls. (**b**) RT-qPCR melt curves of the *Actin* and *β-amylase* genes. (**c**) Comparative expression of *β-amylase* gene relative to *Actin* in wheat seeds collected from plants grown under control and high night temperature (HNT) stress. Tx-Tx86A5606.

Quantitative real-time PCR (RT-qPCR) was performed with the cDNA synthesized from RNA isolated from mature seeds using the modified SDS-LiCl method. Amplification of all the samples with strait amplification curves and Ct values within the range permitted to perform meaningful analysis of expression data (Fig. 5, see Impa et al.^20^ for published expression data on developing seeds using the modified SDS-LiCl method). The expression analysis of 21 genes encoding enzymes involved in the starch metabolism in developing wheat seeds exposed to a range of increasing night temperatures was carried out using the RNA extracted by the modified SDS-LiCl method (See Impa et al.^20^ for published expression data). A single peak in the melt curve analysis and lower standard errors between the Ct values for a gene indicated the specificity of the primers to bind to the cDNA synthesized from the RNA, with no interference of PCR inhibitors (Fig. 5b).

### Modified SDS-LiCl method equally effective on other tissues exposed to different abiotic stresses

**Figure 6:**
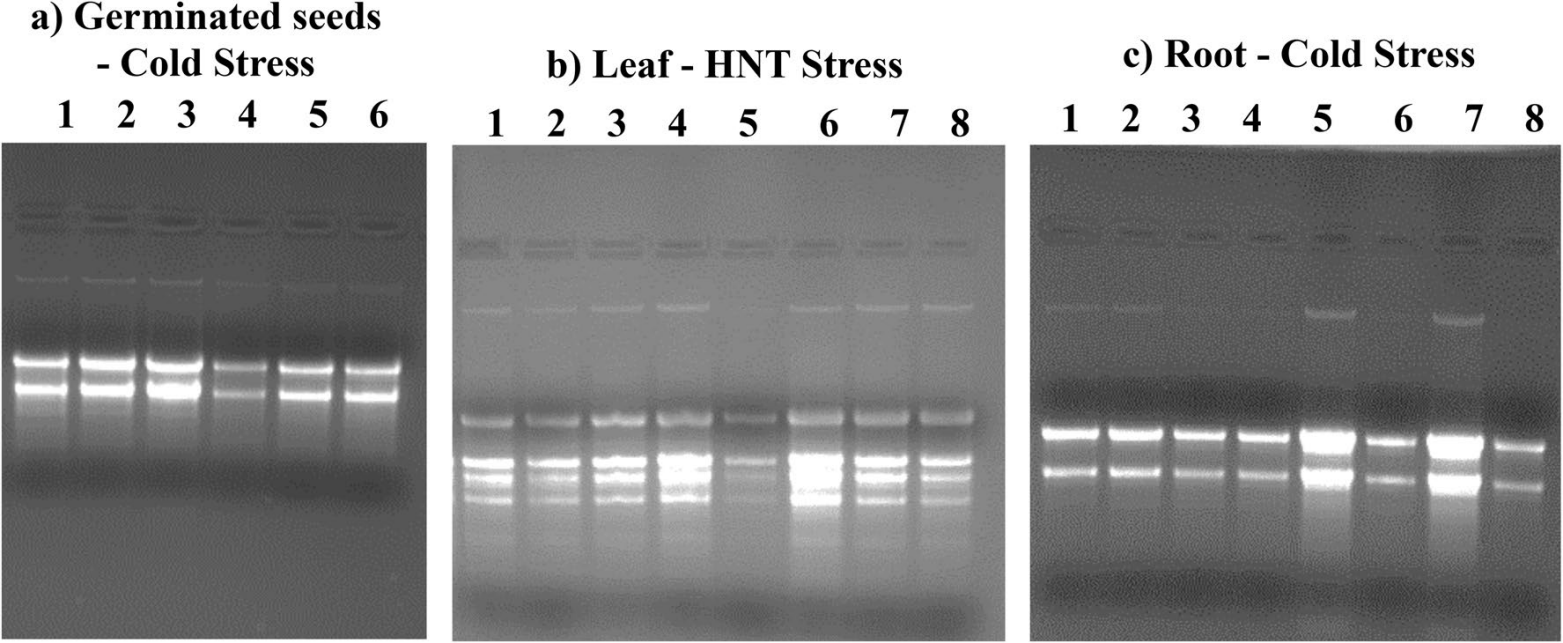
Agarose gel electrophoresis of total RNA extracted using the modified SDS-LiCl extraction method from germinated wheat seeds (**a**), flag leaf (**b**) and root (**c**) tissues exposed to different abiotic stresses. RNA extracted from two genotypes of wheat seedlings germinated under control (30 °C) and cold stress conditions (15 °C) (**a**), Lane 1: Tx- control, Lane 2–3: Tx- cold stress, Lane 4: Tascosa Control, Lane: 5–6 Tascosa Cold stress. RNA isolated from the flag leaves of two wheat genotypes grown under control (26 °C/15 °C) and HNT stress (26 °C/23 °C) (**b**), Lane 1–2: Tx Control, Lane 3–4: Tascosa Control, Lane 5–6: Tx HNT stress, Lane 7–8: Tascosa- HNT stress. RNA extracted from the roots of wheat genotypes grown under control (25 °C) and freezing stress (− 4 °C) (**c**), Lane 1–2: Tx Control, Lane 3–4: Tx Freezing stress, Lane 5–6: Tascosa Control, Lane 7–8: Tascosa Freezing stress. Tx-Tx86A5606. The images of agarose gel electrophoresis used in this figure for the various tissue types were cropped from the original source gel image provided in the supplementary source Fig. 3. The cropped images were auto corrected for the same brightness/contrasts using PowerPoint image tools.

**Figure 7:**
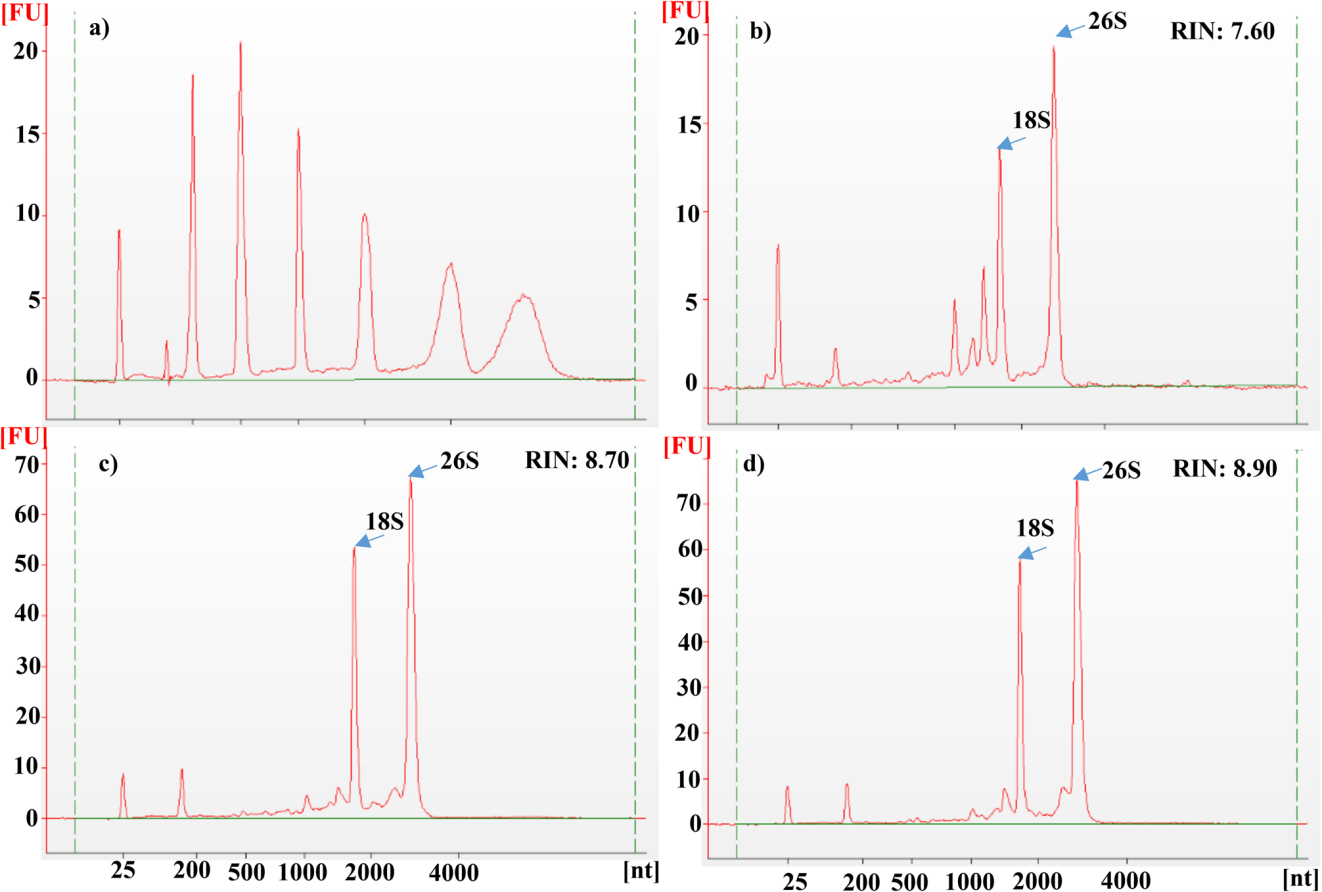
Electropherograms of total RNA isolated from various wheat plant tissues using modified SDS-LiCl method. X-axis units in nt (Nucleotides); Y-axis units in FU (Fluorescence Units). Electropherograms of RNA ladder (**a**), total RNA of wheat flag leaf (**b**), germinated seeds (**c**), and root (**d**).

Further, the modified SDS-LiCl method was used successfully to extract high-quality RNA from germinated wheat seeds, flag leaves, and root tissues that were exposed to cold, freezing and HNT stress. Agarose gel electrophoretic separation and Nanodrop UV spectrophotometer measurements of total RNA from different tissues of wheat genotypes exposed to different abiotic stresses were confirmed for RNA quality with A260/280 and A260/A230 ratios of ≥ 2 and a similar RNA yield to that of control samples (Fig. 6; Table 3; Supplementary Figs. S7–S9). Analysis of RNA samples by Bioanalyzer Agilent 2100 further confirmed high-quality RNA with prominent peaks of rRNA in electropherograms with a higher RIN values (> 7) for various wheat plant tissue RNA isolated by the modified SDS-LiCl method (Fig. 7; Supplementary Figs. S10–S12). Our method reliably provided ample yield and high quality RNA from developing wheat seeds that were exposed to a range of increasing night temperatures (15–27 °C) under controlled environment chambers (Supplementary Figs. S13–S18; Supplementary Table S2; also see Impa et al.^20^ for additional gene expression data). Previous studies have reported decreased concentration of RNA from the samples subjected to abiotic stress^12–14,35^. Similarly, in our study we observed less RNA concentration in the developing grain samples of KS07077M-1 subjected to HNT stress compared to control using the SDS-LiCl method (Supplementary Table S2). However, a similar trend was not observed with the RNA samples extracted using the SDS-LiCl method that were subjected to HNT or other abiotic stresses. Also, RNA extracted using other methods (Ambion TRIZOL, RNeasy Plant Mini Kit (Qiagen), Furtado^6^) did not differ between control and HNT stress samples due to interference of starch. Similarly, an independent study recorded no difference in the RNA yield of wheat leaf tissue subjected to freezing stress^13^. Previous studies have reported that reduced concentration of RNA and interference of secondary metabolites in RNA extraction depends on the severity of stress and tissue age^12^ also the type of tissue^13^. Hence, the lack of a significant impact on RNA concentration in this study could be due to the short duration of exposure under different stresses or due to moderate HNT stress^18^ levels.

**Table 3:** Nano-spectrophotometric analysis of RNA yield, purity and Integrity Numbers (RIN) using Bioanalyzer (Agilent 2100) for the total RNA isolated using the modified SDS-LiCl method from germinated wheat seeds, flag leaf and root tissue exposed to different abiotic stresses.

	RNA yield (μg/100 mg FW)	A260/A280 ratio	A260/A230 ratio	RIN
**Germinated seeds**				
Tx control (30 °C)	33.92 ± 6.96	2.08 ± 0.07	2.18 ± 0.17	7.10
Tx cold stress (15 °C)	37.95 ± 6.80	2.08 ± 0.08	2.32 ± 0.03	8.70
Tascosa control (30 °C)	29.77 ± 3.88	2.17 ± 0.01	2.31 ± 0.03	8.30
Tascosa cold stress (15 °C)	36.69 ± 5.84	2.09 ± 0.08	2.17 ± 0.11	8.40
**Flag leaf**				
Tx control (26/15 °C)	19.53 ± 5.05	2.16 ± 0.03	2.13 ± 0.01	6.90
Tx HNT stress (26/23 °C)	25.17 ± 3.67	2.15 ± 0.04	2.31 ± 0.01	7.00
Tascosa control (26/15 °C)	48.40 ± 5.30	2.18 ± 0.01	2.27 ± 0.11	7.60
Tascosa HNT stress (26/23 °C)	30.66 ± 3.71	2.14 ± 0.04	2.28 ± 0.02	6.90
**Root**				
Tx control (25 °C)	15.00 ± 3.71	2.12 ± 0.01	2.26 ± 0.01	8.90
Tx freezing stress (− 4 °C)	16.79 ± 2.12	2.12 ± 0.05	2.20 ± 0.07	8.10
Tascosa control (25 °C)	48.76 ± 6.96	2.20 ± 0.05	2.15 ± 0.11	7.60
Tascosa freezing stress (− 4 °C)	54.41 ± 7.86	2.11 ± 0.02	2.23 ± 0.09	8.80

Yield and quality were assessed by absorption spectra and ratios of A260/A280 and A260/A230. Tx- Tx86A5606.

^a^Values represent the mean ± SD from at least three technical samples.

Modified SDS-LiCl method also yielded high quality RNA from other cereals seeds (mature and developing maize seeds and developing sorghum seeds) rich in starch, protein, and polyphenols (Supplementary Figs. S19, S20). RNA extraction protocols and commercial kits are limited to specific species and/or tissue^3,17,36^, whereas the modified SDS-LiCl method was suitable for extracting high quality RNA from various crop tissues differing in developmental stages and exposed to different abiotic stresses under controlled environment and field conditions.

In summary, the modified protocol was exclusively developed for extraction of RNA from cereal seed tissue containing high starch, as it is one of the major hurdles challenging functional studies involving developing or mature seeds. This protocol is cost-effective compared to commercially available kits and has been demonstrated to be successful in obtaining quality RNA from mature wheat grains, while TRIZOL, CTAB, and other kits generally failed. The robustness of the modified SDS-LiCl method helped to extract significantly higher yield and quality of RNA from different wheat plant tissues, including, mature, developing and germinated seeds, leaves and roots, exposed to various abiotic stresses, whereas previously published protocols have limited the protocol to either seed tissues of wheat or other cereals^1,2,5,24^ or only leaf tissues^3^. The protocol standardized here has been modified to be a rapid yet reliable, using basic laboratory chemicals that are easily accessible by researchers, whereas previously methods needed more chemicals in the extraction buffer, or expensive reagents kits^1,2,5^. Besides, the versatility of the universal protocol is demonstrated by extracting high-quality RNA from other cereal crop seeds. A comparative assessment including the advantages of the SDS-LiCl method compared to previous protocols is presented in Table 4.

**Table 4:** Comparing SDS-LiCl method with previously published RNA extraction protocols.

	Li and Trick^1^	Wang et al.^2^	Singh et al.^5^	Ambion TRIZOL	RNeasy plant mini kit (Qiagen)	Furtado^6^	CTAB-LiCl	SDS-LiCl method
Type of extraction buffer	SDS + LiCl + GS	SDS + GITC	Tris + GHC	GITC	GHC	GITC + GHC	CTAB	SDS
No. of chemicals in the Ext.Buf	9	6	9	1 (Mix)	1 (Mix)	2 (Mix)	7	6
Requirement of commercial kit reagents	No	Yes	No	Yes	Yes	Yes	No	No
Precipitation chemicals	IPA + NaCl	IPA + SA + Ethanol	SA + Ethanol	IPA	Ethanol	Ethanol	LiCl	LiCl + SA
Tissue types	Seed	Seed	Seed	Wide range but not suitable for seed	Wide range but not suitable for seed	Mature and developing seed	Leaf, shoot, fruit and seed	Mature, developing, and germinated seeds, root and leaf
Tested plant species	Wheat, Rice and Maize	Wheat, Rice, Sorghum and Maize	Wheat, Barley and Maize	Wide range of species	Wide range of species	Wheat	Wheat and 15 other plant species	Wheat, Sorghum and Maize
Time (From extraction until dry pellet)	90 min	160 min	128 min	60 min	30 min	14 h	36 min	62 min

*SDS* sodium dodecyl sulfate, *GITC* guanidine isothiocyanate, *CTAB* cetyltrimethylammonium bromide, *GS* guanidinium sulfate, *GHC* guanidine hydrochloride, *SA* sodium acetate, *IPA* isopropanol, *Ext.Buf.* extraction buffer, *Mix* mixture of chemicals provided in one reagent by the company.

## Conclusion

In conclusion, we have optimized a robust method for extracting high yield and quality RNA from wheat seeds, free from starch and other contaminates. The obtained RNA quality was appropriate for downstream applications demonstrating the effectiveness of the protocol with seeds, that contain high starch and polysaccharides. The modified SDS-LiCl method extracted significantly higher yield and quality of RNA from different wheat tissues, including mature, developing and germinated seeds, leaves and roots exposed to different abiotic stresses. Besides, the versatility of the protocol was further strengthened with high quality RNA extracted from field grown maize and sorghum seeds.

## Supplementary information

Supplementary file1

Supplementary file2

## Data Availability

All related data not presented in the manuscript are made available in the supplementary documents.
