# Supplementary material for: A universal method for high-quality RNA extraction from plant tissues rich in starch, proteins and fiber
Source: Sci Rep. 2020 Oct 9;10:16887. doi: 10.1038/s41598-020-73958-5 (PMC7547072; doi:10.1038/s41598-020-73958-5)
Supplement: Supplementary file 1 — Supplementary file1 [file 41598_2020_73958_MOESM1_ESM.docx]

**Title – A universal method for high-quality RNA extraction from plant tissues rich in starch, proteins and fiber**

**Authors**

Amaranatha R. Vennapusa^1^, Impa M. Somayanda^1^, Colleen J. Doherty^2^ and S. V. Krishna Jagadish^1*^

**Affiliations**

^1^Department of Agronomy, Kansas State University, Manhattan, Kansas 66506, USA.

^2^ Department of Molecular and Structural Biochemistry, North Carolina State University, Raleigh, NC 27695, USA.

**Email addresses**

Amaranatha R. Vennapusa: [amarv@ksu.edu](mailto:amarv@ksu.edu)

Impa M. Somayanda: [impasm@ksu.edu](mailto:impasm@ksu.edu)

Colleen J. Doherty: [cjdohert@ncsu.edu](mailto:cjdohert@ncsu.edu)

S. V. Krishna Jagadish: [kjagadish@ksu.edu](mailto:kjagadish@ksu.edu)

***Corresponding author**

S.V. Krishna Jagadish

Department of Agronomy

2004 Throckmorton Plant Sciences Center,

1712 Claflin Road, Manhattan, Kansas 66506-5501

Tel: + 1 785 706 3263

E-mail - [kjagadish@ksu.edu](mailto:kjagadish@ksu.edu)

ORCID - 0000-0002-1501-0960

**REAGENTS (modified SDS-LiCl):**

- Agarose (molecular biology grade) (Sigma Aldrich, St Louis, MO, cat. no. A9539)
- Bromophenol blue (Sigma Aldrich, St Louis, MO, cat. no. B0126)
- Chloroform (Sigma Aldrich, St Louis, MO, cat. no. CX1056)
- Diethyl pyrocarbonate, DEPC (Sigma Aldrich, St Louis, MO, cat. no. 40718)
- DEPC Nuclease-free water (Millipore Sigma, St Louis, MO, cat. no. 9601-OP)
- Ethanol (EtOH, Sigma Aldrich, St Louis, MO, cat. no. E7023-500ML)
- Ethidium bromide solution (Bio-Rad Laboratories, Hercules, CA, cat. no. 1610433)
- Ethylenediaminetetraacetic acid disodium salt, EDTA 2Na **(**molecular biology grade**)** (Sigma Aldrich, St Louis, MO, cat. no. 324503)
- Glacial acetic acid (Sigma Aldrich, St Louis, MO, cat. no. A6283)
- Iso-amyl alcohol (Sigma Aldrich, St Louis, MO, cat. no. W205710)
- Lithium chloride solution (molecular biology grade) (Sigma Aldrich, St Louis, MO, cat. no. L7026)
- Phenol solution, saturated with 0.1 M citrate buffer, pH 4.3 ± 0.2 (molecular biology grade) (Sigma Aldrich, St Louis, MO, cat. no. P4682)
- Phenol–chloroform- isoamyl alcohol (25: 24:1)
- Polyvinylpyrrolidone, PVP (molecular biology grade) (Sigma Aldrich, St Louis, MO, cat. no. P5288)
- RNase AWAY surface decontaminant (Fisher Scientific, Pittsburgh, PA, cat. no. 21-402-178)
- Sodium acetate anhydrous (molecular biology) (Sigma Aldrich, St Louis, MO, cat. no. S2889)
- Sodium chloride (molecular biology) (Sigma Aldrich, St Louis, MO, cat. no. S3014)
- Sodium dodecyl sulfate, SDS (molecular biology grade) (Sigma Aldrich, St Louis, MO, cat. no. 71725)
- TRIZMA base (BioUltra, molecular biology) (Sigma Aldrich, St Louis, MO, cat. no. 93362)
- β-Mercaptoethanol (molecular biology grade) (Sigma Aldrich, St Louis, MO, cat. no. 63689)

**EQUIPMENT**

- Agarose Gel Electrophoresis Systems (Wide Mini Sub-Cell GT, 15×7cm tray) (Bio-Rad Laboratories, Hercules, CA, cat. no. 1704405)
- Bioanalyzer (Agilent, Santa Clara, CA, Model 2100)
- Prism R Refrigerated Microcentrifuge (Labnet International, Inc, Woodbridge, NJ, C2500-R)
- GEL DOC XR & CHEMIDOC XRS system (Bio-Rad, Hercules, CA, 1708195)
- Nanodrop ND-1000 spectrophotometer (NanoDrop Technologies, Wilmington, DE)
- PowerPac Universal Power Supply (Bio-Rad Laboratories, Hercules, CA, cat. no. 164-5070)

**REAGENT SETUP**

**RNA extraction buffer:** To prepare 100 ml of extraction buffer, add 10 ml of Tris (100 mM final concentration) from 1 M Tris- HCl (pH: 8) stock, 5 ml of EDTA 2Na (25 mM final concentration) from 0.5 M stock, 2.5 g of PVP (2.5%) and 14.61 g of NaCl (2.5 M) then stir with moderate heating (40 ℃) on a stir plate and make up the volume to 97.5 ml with 0.1% DEPC water and autoclave. Add the 2.5 ml of β-Mercaptoethanol (2.5 %) just before use.

**Stock solutions**

**1 M Tris (1000 ml)** Add 121.14 g Tris to 800 ml of DEPC water, adjust the pH to 8 with HCl and make up the volume to 1000 ml with DEPC water.

**0.5 M EDTA (1000 ml)** Add 186.1 g of EDTA 2Na to 800 ml of DEPC water, adjust the pH to 8 using sodium hydroxide pellets, and make up the volume to 1000 ml with DEPC water.

**20% SDS** Add 20 g of SDS to 0.1 % DEPC autoclaved water, stir it on a heated stir plate (60 ℃) to dissolve the SDS, make up the volume to 100 ml and store at room temperature.

**3 M Sodium acetate, pH 4.0** Dissolve 24.6 g of sodium acetate in 50 ml water, adjust the pH to 4.0 with hydrochloric acid and make up the volume to 100 ml with DEPC water.

**Phenol: Chloroform: Isoamyl alcohol (25:24:1)** Mix 25 ml of phenol saturated with 0.1 M citrate buffer, 24 ml of chloroform, and 1 ml of isoamyl alcohol. **CRITICAL STEP** Prepare just before use.

**DEPC-treated water (0.1 %)** Add 1 ml of DEPC to 1000 ml of water. Stir vigorously on the magnetic stir plate to dissolve DEPC in water. Autoclave it to inactivate the DEPC.

**80 % ethanol** Add 80 ml of absolute ethanol to 20 ml of autoclaved DEPC water.

**EQUIPMENT SETUP**

**Ceramic mortar, pestle, glassware, spatula and polypropylene micro centrifuge tubes**

All the glass and plastic materials should be adequately washed with soap and rinsed with water. Treat all the materials with DEPC water and wrap the mortars and pestles with aluminum foil. Autoclave all the plastic and glasswares for 20 min at 121 ℃.

**CRITICAL STEP** Make sure that all the plastic materials are autoclavable and the microcentrifuge tubes can withstand 15,890×g-rpm centrifugal force and endure the phenol-chloroform.

**Microcentrifuge** Wipe the surface of the centrifuge with RNase AWAY and set the temperature to 4 ℃.

Make sure to have access to fume hood, pH meter, magnetic stirrer, heating stir plate, autoclave, vortex mixer, micropipettes, tips, scale, tube racks, -20 ℃ and -80 ℃ freezers, gel electrophorosis apparatus and nano spectrophotometer. **CRITICAL STEP** To avoid contamination with RNases, clean the working area, pipettes, and other materials with RNase-AWAY. Always wear disposable gloves that are free from nucleases. Use sterile plastic and glassware or DEPC water treated materials throughout the experiment.

**STEP by STEP PROCEDURE**

**Tissue collection and homogenization**

**●TIMING** 1 h to 1 h 30 min for eight samples depending on the type of tissue.

1) Immediately transfer the frozen tissue into liquid nitrogen from a -80 ℃ freezer to prevent thawing of samples.

**CRITICAL STEP** Tissue collection is the first important step for isolating quality RNA. Wear disposable gloves and wipe with RNase AWAY (RNase free) solution, also wipe the scissors and other materials used for tissue collection and storage. Tissue should be snap-frozen in liquid nitrogen immediately after collection and stored at -80 °C. Tissue should not be thawed before adding homogenization buffer.

2) Pre-cool the mortar and pestle by pouring liquid nitrogen into them.

3) Grind the tissue (0.12 g) into a fine powder in mortar and pestle using liquid nitrogen.

**CRITICAL STEP** Do not allow the tissue to thaw during grinding. If needed, add more liquid nitrogen while grinding, care needs to be taken while adding more liquid nitrogen to avoid splashing or spilling the tissue out of the mortar, especially while grinding mature or germinated seeds.

**TROUBLESHOOTING**

4) Take 100 mg of ground tissue into a microcentrifuge tube (RNase‐free), add 600 µl RNA extraction buffer to the ground tissue, and mix the sample carefully using a vortex mixer. If tissue does not dissolve well, the extraction buffer volume can be increased up to 1 ml. The excess buffer volume will not affect the RNA yield.

**CRITICAL STEP** Lower amount of buffer decreases the efficiency of extraction; hence, make sure required volume of buffer is added.

5) Incubate the homogenate at room temperature for 5 min and add a final concentration of 2 % SDS (60 μl of 20 % SDS) into the suspension. Vortex the tubes well and incubate at room temperature for 2 min.

**CRITICAL STEP** Avoid keeping the samples in RNA extraction buffer for more than 10 min after adding the SDS.

**TROUBLESHOOTING**

**6)** Centrifuge the homogenate at 15,890×gfor 5 min at 4 ℃.

**RNA extraction:** **●TIMING** 30 min

7) Collect the upper aqueous phase into a new microcentrifuge tube.

**TROUBLESHOOTING**

8) Add equal volume (about 600 µl) of phenol (saturated with 0.1 M citrate buffer): chloroform: isoamyl alcohol (25:24:1) and mix the samples using a vortex mixer.

**CRITICAL STEP** Maintaining the acidic pH is crucial for the separation of DNA and proteins from RNA. Hence, using phenol saturated 0.1 M sodium citrate buffer with pH 4.2 will effectively separate RNA from other contaminants; also, make sure to mix the organic phase thoroughly with the acidic aqueous phase by using a vortex. While mixing, close the tube caps tightly and wear protective gloves, glasses and, clothing.

9) Centrifuge the sample mixture at 15,890×gfor 5 min at 4 ℃.

10) Collect the upper aqueous phase.

**CRITICAL STEP** The upper aqueous phase should be carefully collected without disturbing the lower layers, which contains proteins, polysaccharides, and organic compounds.

**TROUBLESHOOTING**

11) Add chloroform (200 μl) and mix the sample well using a vortex mixer.

12) Centrifuge at 15,890×g for 5 min at 4 ℃.

13) Collect the upper aqueous phase.

**RNA precipitation: ●TIMING** 25 min

14) Add about 160 µl of 8 M LiCl to a final concentration of 2 M, and 1/10 volume of 3 M sodium acetate (pH 4.8, 60 μl) and mix by gentle inversions; four to six times.

**CRITICAL STEP** Make sure to mix samples thoroughly after adding each chemical to enhance the RNA precipitation efficiency.

**TROUBLESHOOTING**

15) Incubate the sample at -20 ℃ for 15 min for RNA precipitation.

**PAUSE POINT** The samples can be stored overnight or 24 h at -20 ℃. Overnight incubation enhances the yield.

**RNA washing ●TIMING** 30 min

16) Centrifuge the sample at 15,890×g for 5 min at 4 ℃.

17) Discard the supernatant and wash the transparent pellet with the 500 μl of 2 M LiCl by centrifuging at 15,890×g for 5 min at 4 ℃.

**CRITICAL STEP** Remove the supernatant carefully without losing the RNA pellet, as the transparent pellet may not be clearly visible sometimes.

18) Decant the supernatant, and again, wash the pellet with pre-chilled 80 % ethanol by centrifuging at 15,890×g for 5 min at 4 ℃.

**PAUSE POINT** The samples can be stored at -20 ℃ for 3 months.

**Dissolving RNA** **●TIMING** 20 min

19) Decant the wash solution and evaporate the residual ethanol under laminar airflow hood and allow the pellet to dry (but do not over dry).

20) Dissolve the RNA pellet in DEPC‐Water with an appropriate volume (30-50 μl) and store at -80 ℃ for downstream applications.

**PAUSE POINT** The samples can be stored at -20 °C for short-term or -80 ℃ for long-term storage. Avoid repeated freeze-thaw cycles of the RNA stock.

**●TIMING**

Steps 1-6: Tissue collection and homogenization, 1 h to 1h 30 min for eight samples depending on the tissue. Leaf and root-1 h, developing and mature or germinated seeds - 1 h 30 min.

Steps 7-13: RNA extraction, 30 min

Steps 14 and 15: RNA precipitation including centrifugation, 25 min

Steps 16-18: RNA washing, 30 min

Steps 19 and 20: Dissolving RNA, 20 min

**TROUBLESHOOTING**

| **Step** | **Problem** | **Cause** | **Solution** |
| --- | --- | --- | --- |
| 3 | Splashing/spilling of seed tissue | Liquid nitrogen makes the seeds (developing, mature or germinated) seeds very hard and slippery in the mortar | Use bigger mortar, pestles, and more liquid nitrogen. Crush the samples carefully first instead of grinding directly. Ensure to add liquid nitrogen slowly to prevent tissue spilling out of the mortar. |
| 5 | Solidification | Adding an insufficient amount of RNA extraction buffer for homogenizing the tissue. | Increase the volume of extraction buffer by up to 1 ml |
|  |  | Clumps formation in case of improper mixing of extraction buffer and the homogenate | Homogenate should be thoroughly mixed before and after adding the SDS |
|  |  | SDS is not thoroughly mixed | Incubation period can be increased by >2 min after adding SDS |
| 7 | Viscous substance in the aqueous phase | Improper separation of layers | If viscosity persists in the supernatant after centrifugation, extend the centrifugation period from 7-10 min |
| 10 | Contamination of polysaccharides and proteins | Pipetting organic phase into the aqueous phase | Collect the upper aqueous phase cautiously without contaminating the extract with the lower organic layer, which contains a lot of starch and proteins that may interfere with the quality of the RNA. To overcome this, leave a small layer of solution at an intermediate phase that avoids pipetting the lower phase |
| 14 | Lower yield | Low volume of the aqueous phase | Collect adequate amount of aqueous phase at each phase separation step for precipitation of RNA |
|  |  | Inadequate mixing of chemicals after adding to the aqueous solution | LiCl and Sodium acetate should be thoroughly mixed with the aqueous phase after adding each chemical for maximum precipitation of RNA |
| 20 | DNA contamination | The chemicals used in this procedure could extract DNA along with RNA | Perform DNAse I treatment before using the RNA for downstream applications |

**Reagents used in the other RNA extraction protocols**

**TRIZOL Method**

TRIZOL Reagent (Ambion TRIZOL Reagent Kit (by Life Technologies, now its Invitrogen, Carlsbad, CA, cat. no. 15596-026)

Chloroform (Sigma Aldrich, St Louis, MO, cat. no. CX1056)

Isopropanol (IPA-molecular biology grade, Sigma Aldrich, St Louis, MO, cat. no. I9516-500ML)

RNase-free water (DEPC Nuclease-free water, Millipore Sigma, St Louis, MO, cat. no. 9601)

Ethanol (EtOH, Sigma Aldrich, St Louis, MO, cat. no. E7023-500ML)

**RNeasy Plant Mini Kit**

RNeasy Plant Mini Kit (Cat# 74903, Qiagen, USA).

Buffer RLC (guanidine hydrochloride)

Buffer RW1

Buffer RPE

β-Mercaptoethanol (β-ME, molecular biology grade, Sigma Aldrich, St Louis, MO, cat. no. 63689)

100% Ethanol (EtOH, Sigma Aldrich, St Louis, MO, cat. no. E7023-500ML)

RNAse-free water (DEPC Nuclease-free water, Millipore Sigma, St Louis, MO, cat. no. 9601)

**Furtado (2014)^6^ method**

RNeasy Plant Mini Kit (Cat# 74903, Qiagen, USA).

Buffer RW1

Buffer RPE

Chloroform (Sigma Aldrich, St Louis, MO, cat. no. CX1056)

96–100% Ethanol (EtOH, Sigma Aldrich, St Louis, MO, cat. no. E7023-500ML)

RNAse-free water (DEPC Nuclease-free water, Millipore Sigma, St Louis, MO, cat. no. 9601)

**CTAB-LiCl method**

3% β-Mercaptoethanol (molecular biology grade, Sigma Aldrich, St Louis, MO, cat. no. 63689)

Chloroform-isoamylalcohol (24:1)

Chloroform (Sigma Aldrich, St Louis, MO, cat. no. CX1056)

2% CTAB- Cetyltrimethylammonium bromide (molecular biology grade, Fisher Scientific, Pittsburgh, PA, cat. no. ICN19400480)

DEPC (Diethyl dicarbonate, Sigma Aldrich, St Louis, MO, cat. no. 40718)

70% and 95% ethanol (EtOH, Sigma Aldrich, St Louis, MO, cat. no. E7023-500ML)

25 mM Ethylenediaminetetraacetic acid (EDTA, Sigma Aldrich, St Louis, MO, cat. no. 324503)

Iso-amyl alcohol (Sigma Aldrich, St Louis, MO, cat. no. W205710)

8 M Lithium chloride (LiCl-Sigma Aldrich, St Louis, MO, cat. no. L7026)

2% Polyvinylpyrrolidone (PVP, Sigma Aldrich, St Louis, MO, cat. no. P5288)

2 M Sodium chloride (NaCl, molecular biology, Sigma Aldrich, St Louis, MO, cat. no. S3014)

3 M Sodium acetate (pH 5.5), (NaOAc, Sigma Aldrich, St Louis, MO, cat. no. S2889)

0.5 g/L Spermidine (molecular biology grade, Sigma Aldrich, St Louis, MO, cat. no. S0266)

100 mM Tris-HCl (pH 8.0) (molecular biology grade, Sigma Aldrich, St Louis, MO, cat. no. 93363)
