# Supplementary material for: A universal method for high-quality RNA extraction from plant tissues rich in starch, proteins and fiber
Source: Sci Rep. 2020 Oct 9;10:16887. doi: 10.1038/s41598-020-73958-5 (PMC7547072; doi:10.1038/s41598-020-73958-5)
Supplement: Supplementary file 2 — Supplementary file2 [file 41598_2020_73958_MOESM2_ESM.docx]

**Title – A universal method for high-quality RNA extraction from plant tissues rich in starch, proteins and fiber**

**Authors**

Amaranatha R. Vennapusa^1^, Impa M. Somayanda^1^, Colleen J. Doherty^2^ and S. V. Krishna Jagadish^1*^

**Affiliations**

^1^Department of Agronomy, Kansas State University, Manhattan, Kansas 66506, USA.

^2^ Department of Molecular and Structural Biochemistry, North Carolina State University, Raleigh, NC 27695, USA.

**Email addresses**

Amaranatha R. Vennapusa: [amarv@ksu.edu](mailto:amarv@ksu.edu)

Impa M. Somayanda: [impasm@ksu.edu](mailto:impasm@ksu.edu)

Colleen J. Doherty: [cjdohert@ncsu.edu](mailto:cjdohert@ncsu.edu)

S. V. Krishna Jagadish: [kjagadish@ksu.edu](mailto:kjagadish@ksu.edu)

***Corresponding author**

S.V. Krishna Jagadish

Department of Agronomy

2004 Throckmorton Plant Sciences Center,

1712 Claflin Road, Manhattan, Kansas 66506-5501

Tel: + 1 785 706 3263

E-mail - [kjagadish@ksu.edu](mailto:kjagadish@ksu.edu)

ORCID - 0000-0002-1501-0960


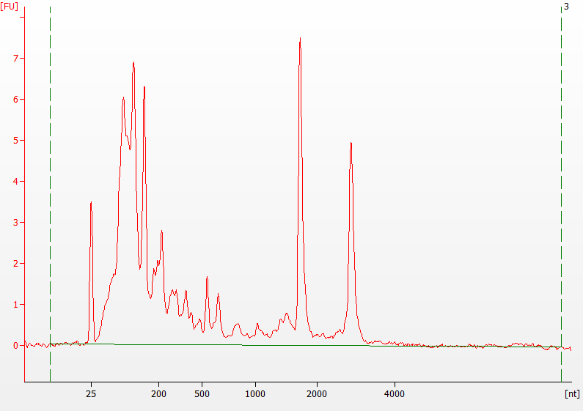


**c) Tascosa HNT stress**

**500**

**25**

**2000**

**5**

**4**

**3**

**2**

**1**

**[FU]**

**0**

**6**

**7**

**200**

**1000**

**4000**

**RIN: 6.50**

**18S**

**26S**

**[nt]**


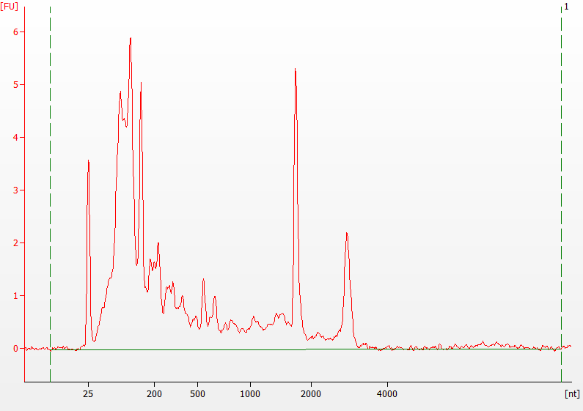


**a) Tascosa control**

**5**

**4**

**3**

**2**

**1**

**[FU]**

**0**

**6**

**18S**

**26S**

**RIN: 5.50**


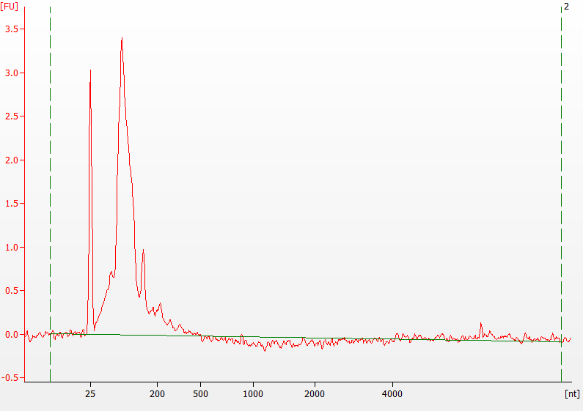


**b) Tx-control**

**3.0**

**2.5**

**2.0**

**1.5**

**1.0**

**0.5**

**0.0**

**-0.5**

**3.5**

**[FU]**

**RIN: 2.60**


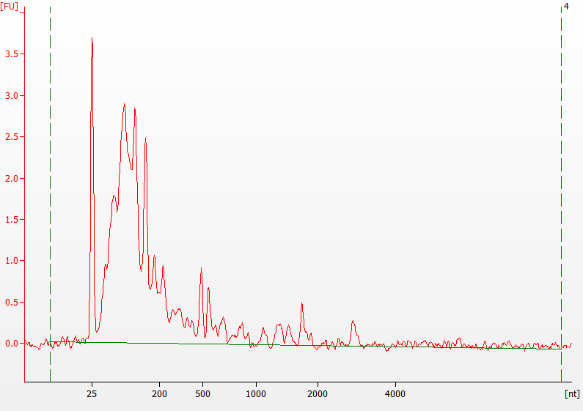


**d) Tx- HNT stress**

**200**

**25**

**1000**

**2000**

**4000**

**500**

**3.0**

**2.5**

**2.0**

**1.5**

**1.0**

**0.5**

**0.0**

**3.5**

**[FU]**

**RIN: 2.70**

**18S**

**26S**

**[nt]**

**Supplementary Figure 1.** Electropherograms generated from mature wheat seed RNA isolated using Ambion TRIZOL method. Mature seed samples were collected from wheat plants grown under control and HNT stress in field. X –axis units in nt (Nucleotides); Y –axis units in FU (Fluorescence Units). Tx -Tx86A5606, HNT – High night temperature.


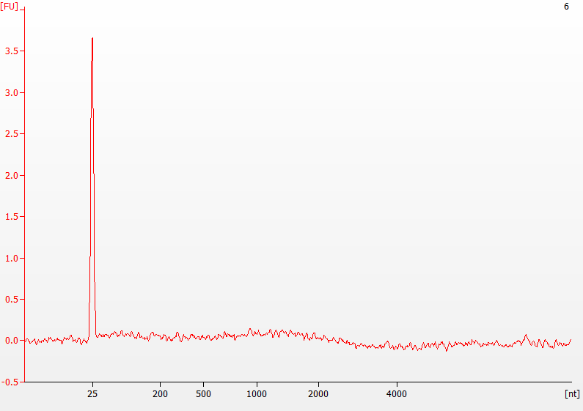


**b) Tx-control**

**3.0**

**2.5**

**2.0**

**1.5**

**1.0**

**0.5**

**0.0**

**-0.5**

**3.5**

**[FU]**

**RIN: N/A**


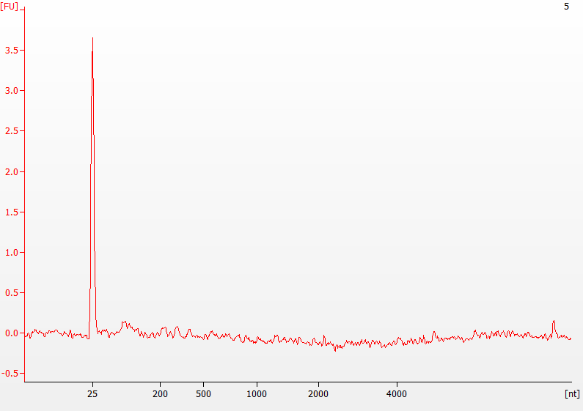


**a) Tascosa control**

**3.0**

**2.5**

**2.0**

**1.5**

**1.0**

**0.5**

**0.0**

**-0.5**

**3.5**

**[FU]**

**RIN: N/A**


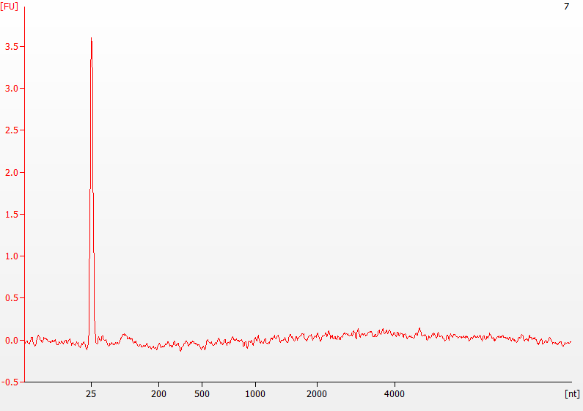


**c) Tascosa HNT stress**

**3.0**

**2.5**

**2.0**

**1.5**

**1.0**

**0.5**

**0.0**

**-0.5**

**3.5**

**[FU]**

**RIN: N/A**

**500**

**25**

**2000**

**200**

**1000**

**4000**

**[nt]**


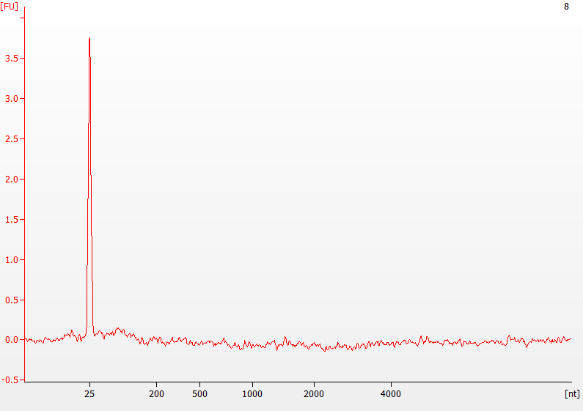


**d) Tx- HNT stress**

**3.0**

**2.5**

**2.0**

**1.5**

**1.0**

**0.5**

**0.0**

**-0.5**

**3.5**

**[FU]**

**RIN: N/A**

**500**

**25**

**2000**

**200**

**1000**

**4000**

**[nt]**

**Supplementary Figure 2.** Electropherograms generated from mature wheat seed RNA isolated using RNeasy Plant Mini Kit (Qiagen) method. Mature seeds were collected from wheat plants grown under control and HNT stress in field. X –axis units in nt (Nucleotides); Y –axis units in FU (Fluorescence Units). Tx -Tx86A5606, HNT – High night temperature.


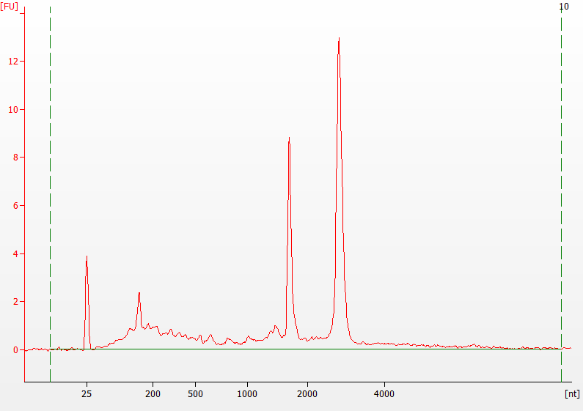


**4**

**2**

**[FU]**

**0**

**6**

**8**

**10**

**12**

**RIN: 8.10**

**18S**

**26S**

**b) Tx-control**


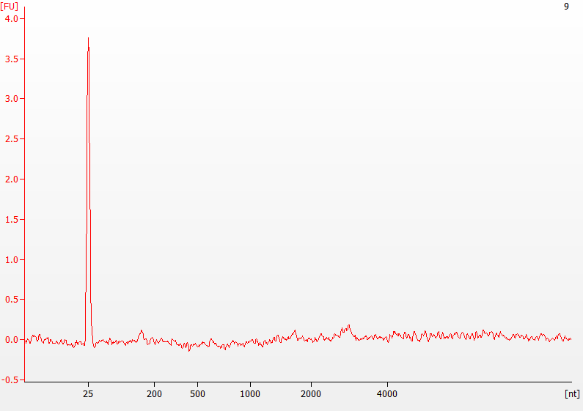


**3.0**

**2.5**

**2.0**

**1.5**

**1.0**

**0.5**

**0.0**

**-0.5**

**3.5**

**[FU]**

**4.0**

**RIN: N/A**

**a) Tascosa control**


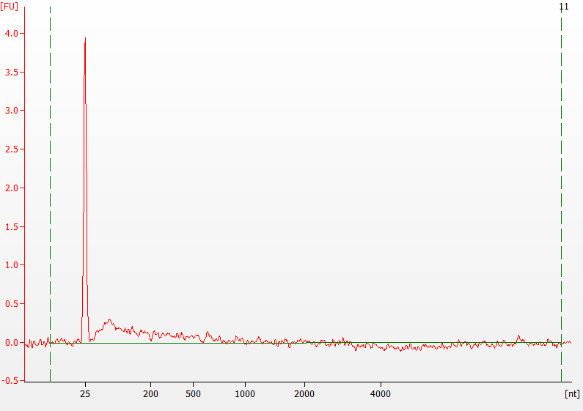


**3.0**

**2.5**

**2.0**

**1.5**

**1.0**

**0.5**

**0.0**

**-0.5**

**3.5**

**[FU]**

**4.0**

**500**

**25**

**2000**

**200**

**1000**

**4000**

**RIN: N/A**

**[nt]**

**c) Tascosa HNT stress**


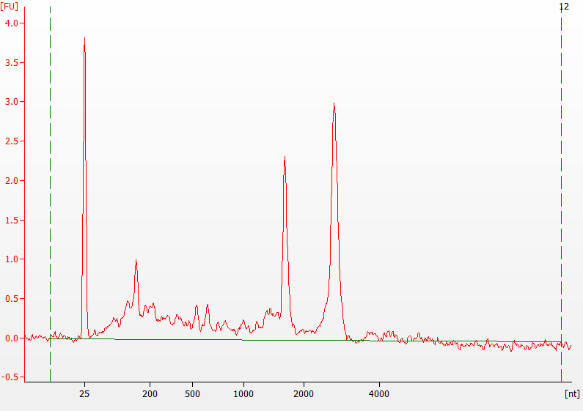


**3.0**

**2.5**

**2.0**

**1.5**

**1.0**

**0.5**

**0.0**

**-0.5**

**3.5**

**500**

**25**

**2000**

**200**

**1000**

**4000**

**18S**

**26S**

**[nt]**

**[FU]**

**4.0**

**d) Tx- HNT stress**

**RIN: 7.40**

**Supplementary Figure 3.** Electropherograms generated from mature wheat seed RNA isolated using Furtado, 2014^6^ method. Mature seed were collected from wheat plants grown under control and HNT stress in field. X –axis units in nt (Nucleotides); Y –axis units in FU (Fluorescence Units). Tx -Tx86A5606, HNT – High night temperature.


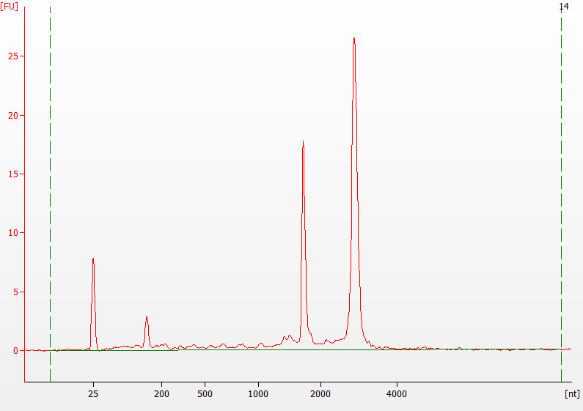


**25**

**20**

**15**

**10**

**5**

**0**

**[FU]**

**RIN: 9.30**

**18S**

**26S**

**b) Tx-control**


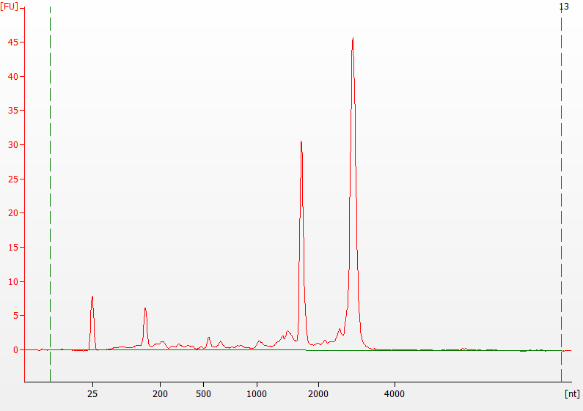


**25**

**20**

**15**

**10**

**5**

**0**

**[FU]**

**35**

**30**

**45**

**40**

**RIN: 9.00**

**18S**

**26S**

**a) Tascosa control**


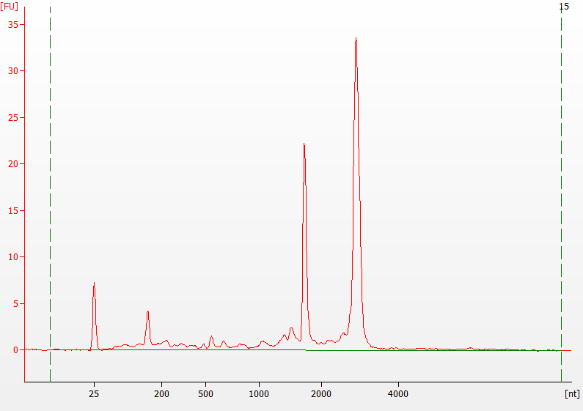


**25**

**20**

**15**

**10**

**5**

**0**

**[FU]**

**35**

**30**

**500**

**25**

**2000**

**200**

**1000**

**4000**

**RIN: 8.90**

**18S**

**26S**

**[nt]**

**c) Tascosa HNT stress**


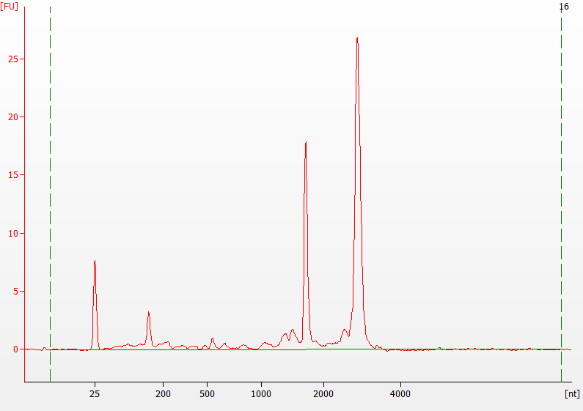


**25**

**20**

**15**

**10**

**5**

**0**

**[FU]**

**500**

**25**

**2000**

**200**

**1000**

**4000**

**RIN: 9.10**

**18S**

**26S**

**[nt]**

**d) Tx- HNT stress**

**Supplementary Figure 4.** Electropherograms generated from mature wheat seed RNA isolated using CTAB-LiCl method. Mature seed samples were collected from wheat plants grown under control and HNT stress in field. X –axis units in nt (Nucleotides); Y –axis units in FU (Fluorescence Units). Tx -Tx86A5606, HNT – High night temperature.


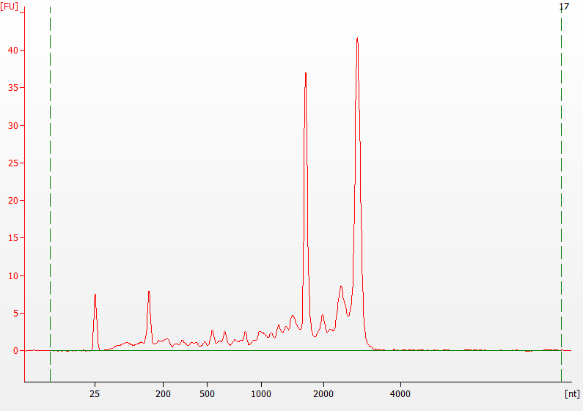


**25**

**20**

**15**

**10**

**5**

**0**

**[FU]**

**35**

**30**

**40**

**RIN: 7.60**

**18S**

**26S**

**a) Tascosa control**


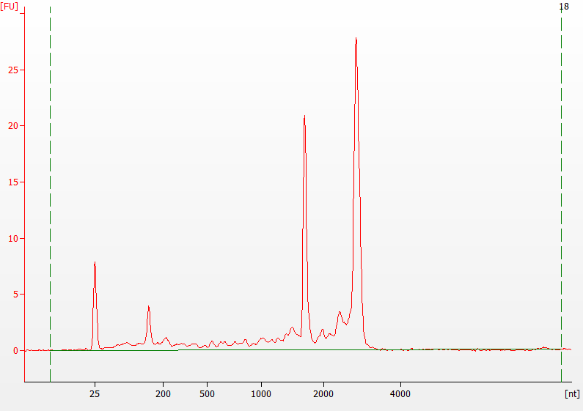


**25**

**20**

**15**

**10**

**5**

**0**

**[FU]**

**RIN: 8.40**

**18S**

**26S**

**b) Tx-control**


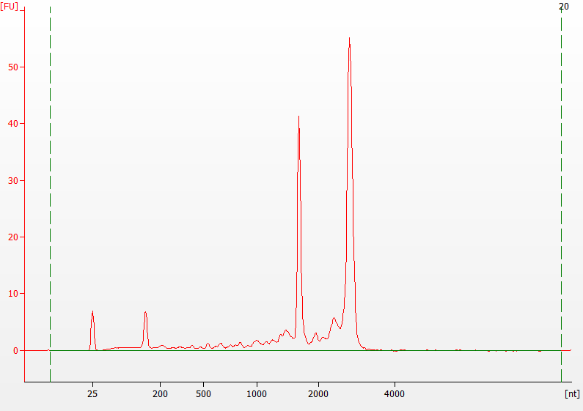


**500**

**25**

**2000**

**200**

**1000**

**4000**

**[nt]**

**20**

**10**

**0**

**[FU]**

**30**

**40**

**50**

**RIN: 8.80**

**18S**

**26S**

**d) Tx- HNT stress**


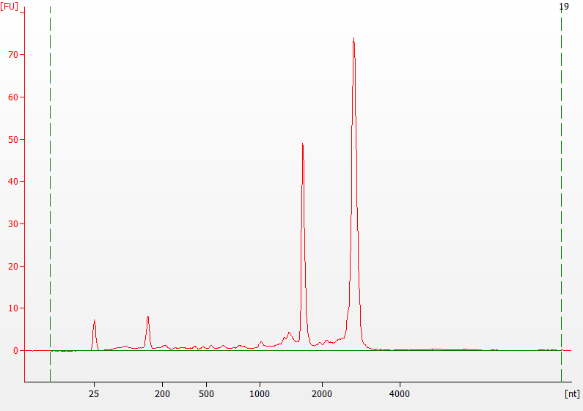


**500**

**25**

**2000**

**200**

**1000**

**4000**

**[nt]**

**20**

**10**

**0**

**[FU]**

**30**

**40**

**50**

**60**

**70**

**RIN: 9.10**

**18S**

**26S**

**c) Tascosa HNT stress**

**Supplementary Figure 5.** Electropherograms generated from mature wheat seed RNA isolated using modified SDS-LiCl method. Mature seed samples were collected from wheat plants grown under control and HNT stress in field. X –axis units in nt (Nucleotides); Y –axis units in FU (Fluorescence Units). Tx -Tx86A5606, HNT – High night temperature.

`


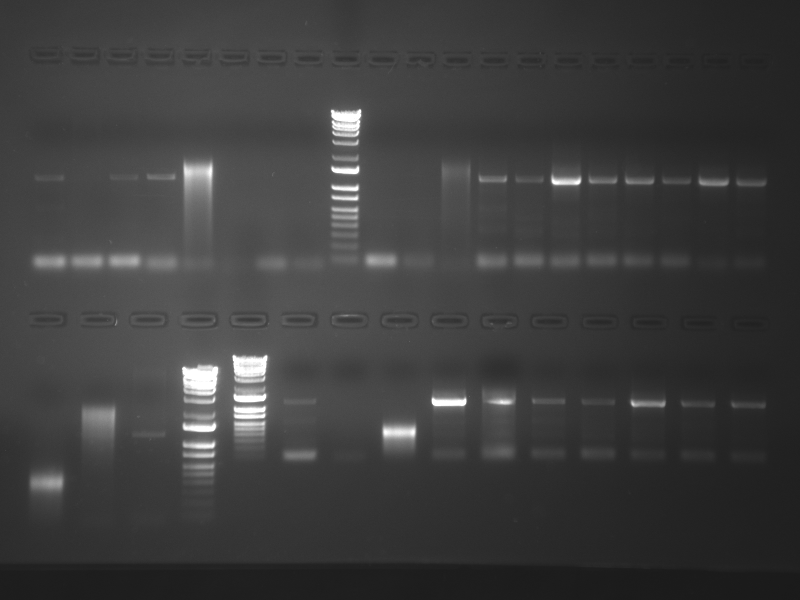


**b) RNeasy Plant Mini Kit-Qiagen**

**1 2 3**

**1 2 3 4**

**1 2 3 4**

**a) Ambion**

**TRIZOL**

**e) Modified**

**SDS-LiCl**

**d) CTAB-LiCl**

**c) Furtado,**

**2014**

**Ladder**

**1 2 3 4**

**1 2 3 4**

1.2 Kb

**Supplementary Figure 6:** PCR amplification of EPSPS gene fragment with a size of 1229 bp (1.2 Kb) using cDNA synthesized from the mature wheat seed of total RNA extracted following Ambion TRIZOL (a), RNeasy Plant Mini Kit (Qiagen) (b), Furtado, 2014^6^ (c), CTAB-LiCl (d), and modified SDS-LiCl methods. Total RNA was extracted from mature wheat seeds collected from plants grown under ambient and post-flowering high night temperature (HNT) stress conditions in the field (Hein et al.^18^). The total RNA from wheat seeds of four different genotypes are numerically labeled from 1 to 4. 1: Tx Control, 2: Tx HNT stress, 3: Tascosa Control and 4: Tascosa HNT stress. Tx- Tx86A5606. Ladder- 100 bp to10Kb of Logic DNA ladder (Lamda Biotech, St. Louis, MO, USA).

**Supplementary Table 1:** Mean and standard error of raw quantification cycle (Cq) values of the mature wheat seed samples cDNA synthesized from total RNA samples isolated using five different protocols. The total RNA was extracted using five different RNA isolation protocols, and cDNA was synthesized using RevertAid First Strand cDNA Synthesis Kit (Thermo Scientific, MA, USA). The cDNA used to perform RT-qPCR and Cq values were recorded using CFX Manager Software version 3.1 (Bio-Rad, CA, USA). The control samples were collected from plants grown under ambient temperature, while high night temperature (HNT) stress samples were collected from plants grown under custom-built heat tents with +3.2 ℃ higher night temperature than ambient temperature (22.7 ^o^C) during grain filling (Hein et al.^18^). Values presented are the mean ± standard error of three technical replicate samples. Tx-Tx86A5606, BMY- Beta-amylase.

| **Sample name** | **Mean Cq ± SEM values** | | | | | |
| --- | --- | --- | --- | --- | --- | --- |
|  | **Gene name** | **Ambion TRIZOL** | **RNeasy Plant Mini Kit-Qiagen** | **Furtado, 2014^6^** | **CTAB-LiCl** | **Modified**  **SDS-LiCl** |
| Tx Control | *Actin* | 26.40 ± 0.34 | 36.87 ± 0.66 | 26.40 ± 0.34 | 22.86 ± 0.24 | 21.18 ± 0.12 |
| Tx HNT | *Actin* | 28.49 ± 0.13 | 34.28 ± 0.30 | 28.49 ± 0.13 | 23.48 ± 0.19 | 21.33 ± 0.05 |
| Tascosa Control | *Actin* | 27.87 ± 0.13 | 33.09 ± 0.23 | 27.87 ± 0.13 | 23.70 ± 0.09 | 21.99 ± 0.02 |
| Tascosa HNT | *Actin* | 27.09 ± 0.23 | 33.32 ± 0.30 | 27.09 ± 0.23 | 23.34 ± 0.00 | 21.45 ± 0.11 |
| Tx Control | *BMY* | 22.18 ± 0.15 | 28.57 ± 10.0 | 22.18 ± 0.15 | 19.55 ± 0.82 | 18.32 ± 0.11 |
| Tx HNT | *BMY* | 26.95 ± 1.13 | 28.47 ± 3.52 | 26.95 ± 1.13 | 17.71 ± 0.24 | 17.40 ± 0.29 |
| Tascosa Control | *BMY* | 27.39 ± 4.37 | 26.77 ± 4.81 | 27.39 ± 4.37 | 18.36 ± 0.04 | 17.41 ± 0.28 |
| Tascosa HNT | *BMY* | 25.51 ± 1.42 | 29.18 ± 3.54 | 25.51 ± 1.42 | 18.57 ± 0.19 | 17.42 ± 0.13 |


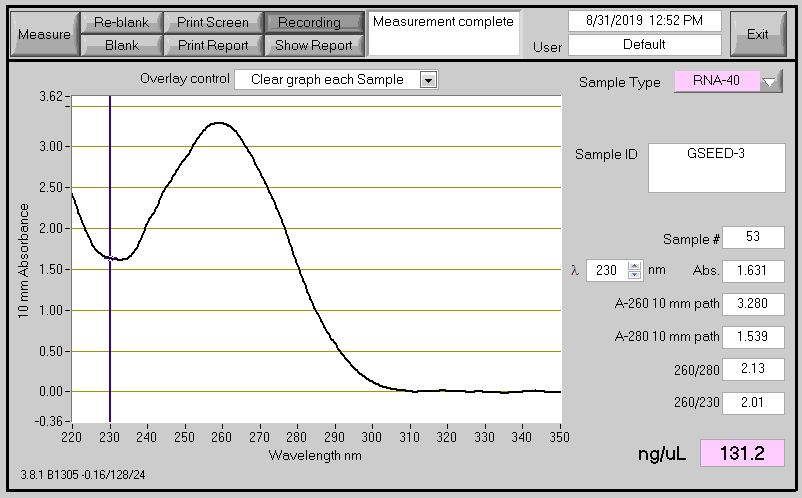


**b)**


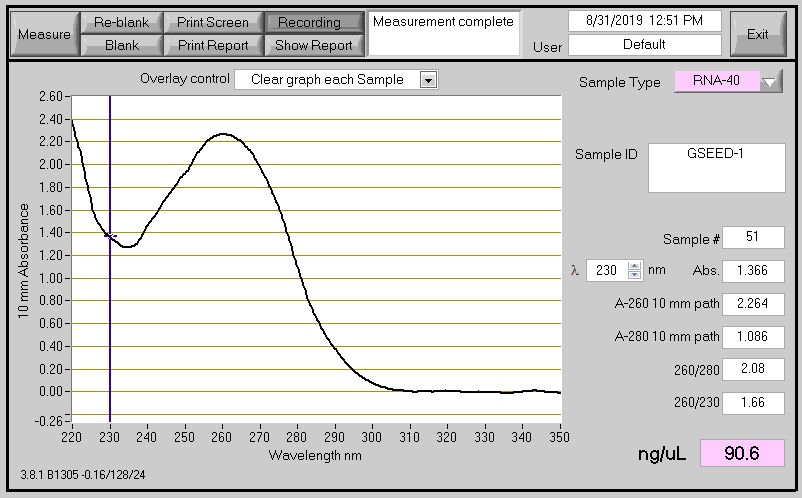


**a)**


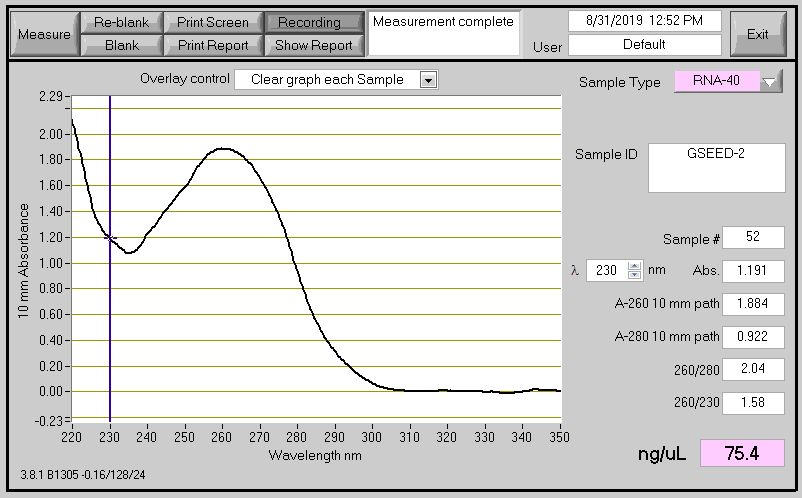


**d)**


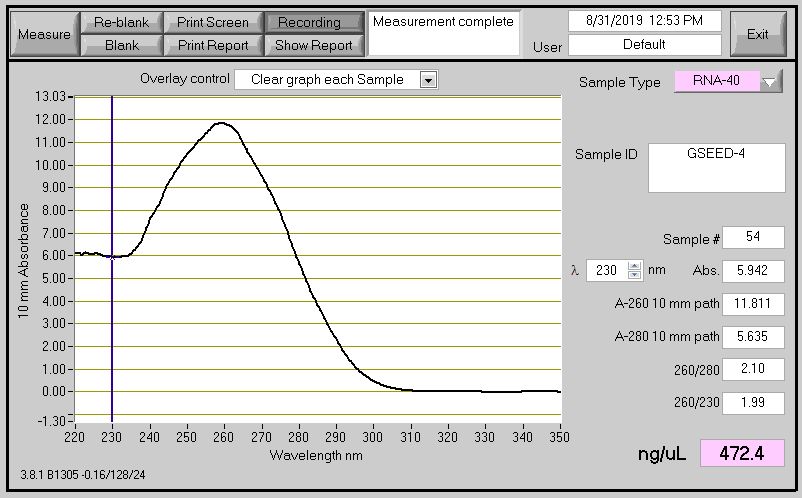


**c)**

**Supplementary Figure 7.** Absorbance spectrum of the total RNA extracted using modified SDS-LiCl method from germinated wheat seeds exposed to control (30 ℃) and cold stress (15 ℃). Tx Control (a), Tascosa Control (b), Tx Cold stress (c) and Tascosa Cold stress (d). The broad absorbance peak at 260 nm and no other peaks at 230 nm and 280 nm indicates the improved yield and quality of the RNA. Tx- Tx86A5606.


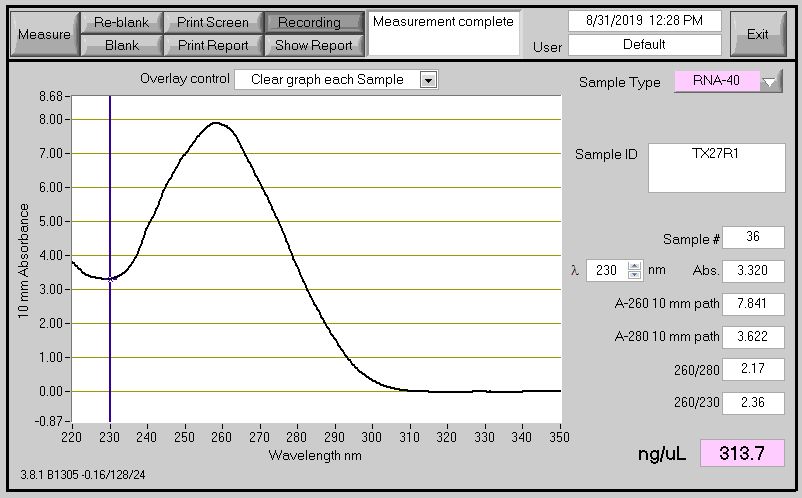


**d)**


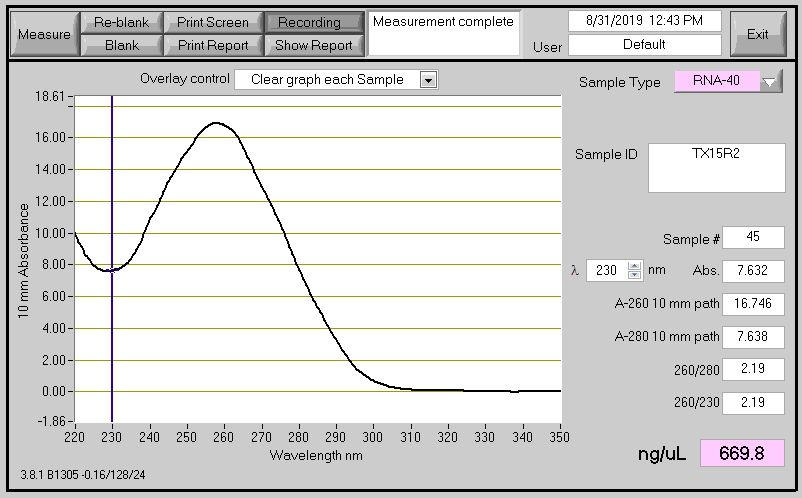


**c)**


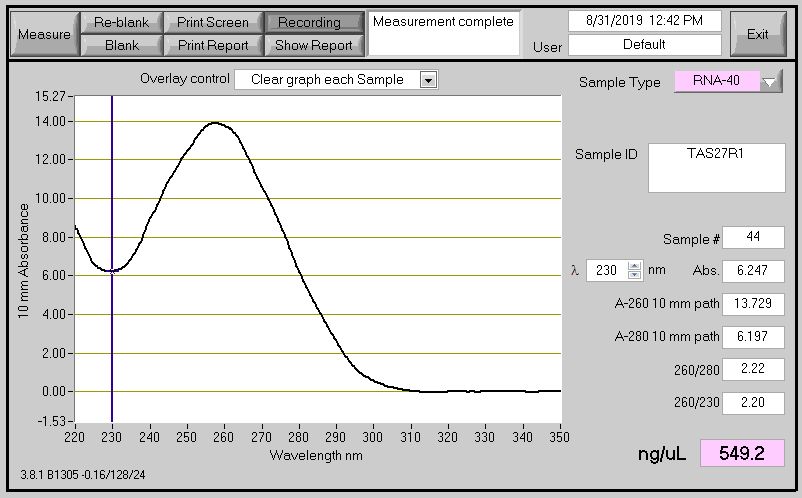


**b)**


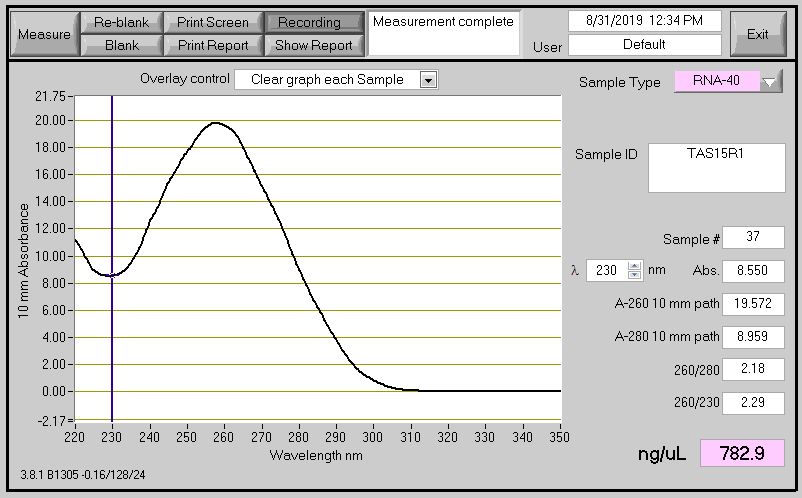


**a)**

**Supplementary Figure 8.** Absorbance spectrum of the total RNA isolated using modified SDS-LiCl method from flag leaves of wheat genotypes grown under control (26 ℃/15 ℃) and HNT stress (26 ℃/23 ℃). Tx Control (**a)**, Tascosa Control **(b)**, Tx HNT stress **(c)** and Tascosa HNT stress **(d)**. The broad absorbance peak at 260 nm and no other peaks at 230 nm and 280 nm indicates the improved yield and quality of the RNA. Tx- Tx86A5606, HNT – High night temperature.


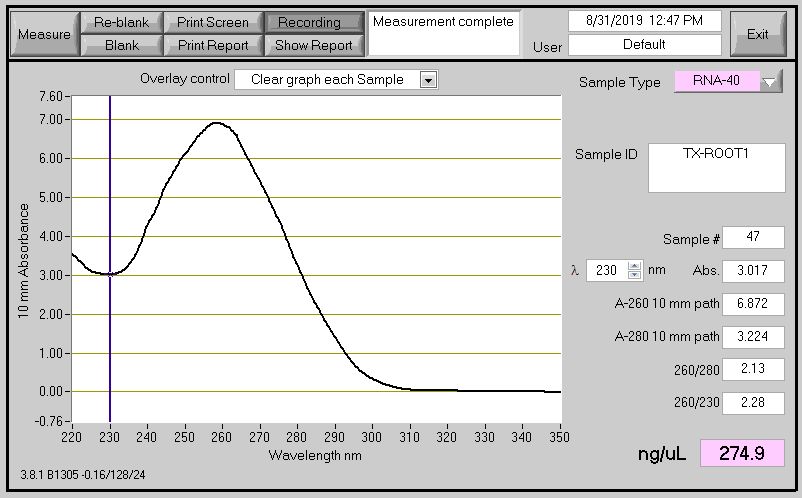


**c)**


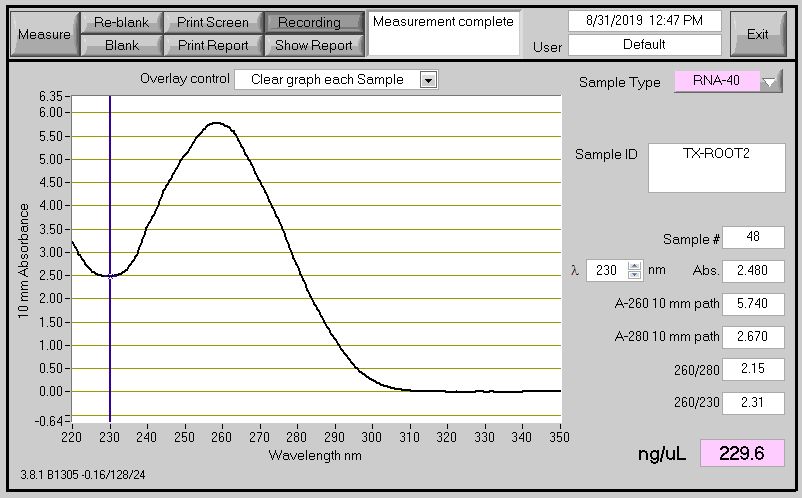


**d)**


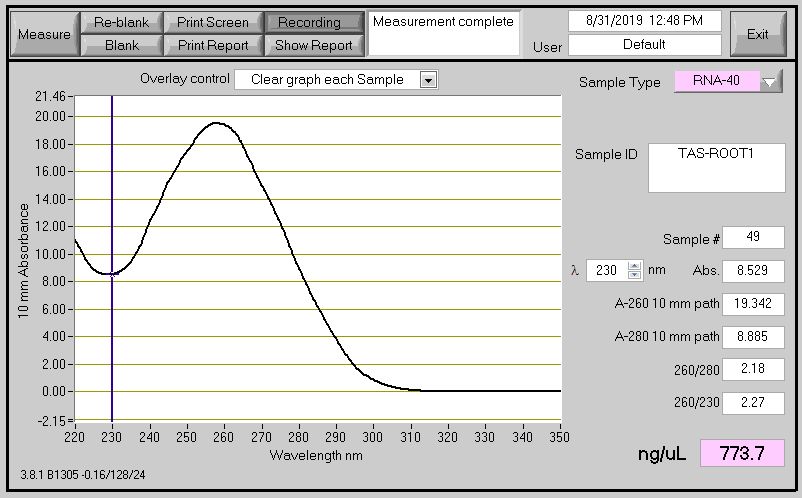


**a)**


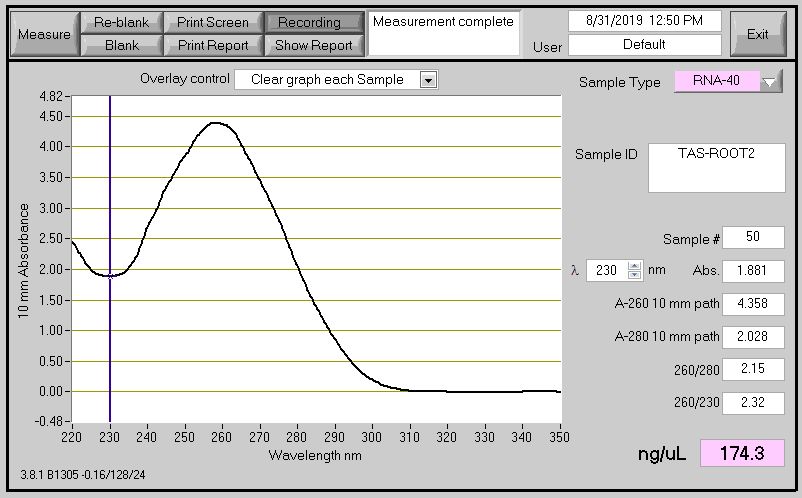


**b)**

**Supplementary Figure 9.** Absorbance spectrum of the total RNA isolated using modified SDS-LiCl method from roots of wheat genotypes grown under control (25 ℃) and freezing stress (-4 ℃), Tx Control **(a)**, Tascosa Control **(b)**, Tx Freezing stress **(c)** and Tascosa Freezing stress **(d)**. The broad absorbance peak at 260 nm and no other peaks at 230 nm and 280 nm indicates the improved yield and quality of the RNA. Tx- Tx86A5606.


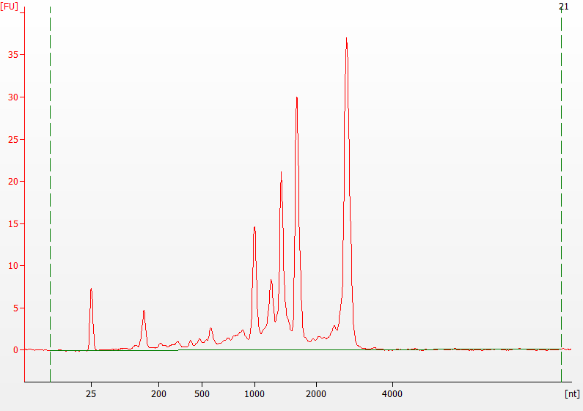


**25**

**20**

**15**

**10**

**5**

**0**

**[FU]**

**35**

**30**

**RIN: 6.90**

**18S**

**26S**

**a) Tx-leaf- control**

**[nt]**


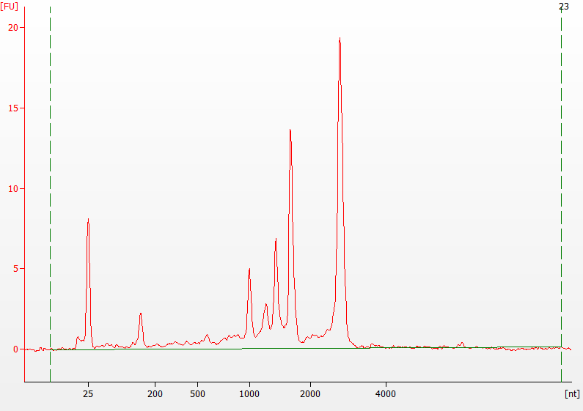


**20**

**15**

**10**

**5**

**0**

**[FU]**

**500**

**25**

**2000**

**200**

**1000**

**4000**

**RIN: 7.60**

**18S**

**26S**

**c) Tascosa-leaf- control**


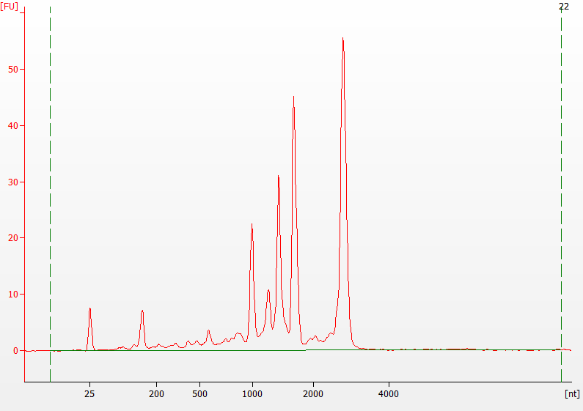


**20**

**10**

**0**

**30**

**40**

**50**

**[FU]**

**RIN: 7.00**

**18S**

**26S**

**b) Tx-leaf- HNT stress**

**[nt]**


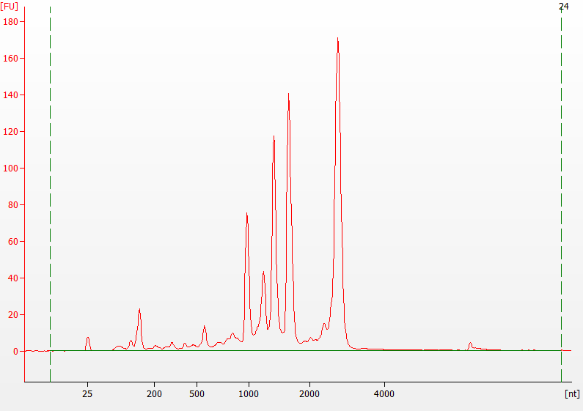


**20**

**0**

**[FU]**

**40**

**60**

**80**

**100**

**120**

**140**

**160**

**180**

**500**

**25**

**2000**

**200**

**1000**

**4000**

**RIN: 6.90**

**18S**

**26S**

**d) Tascosa-leaf- HNT stress**

**Supplementary Figure 10.** Electropherograms of total RNA isolated using modified SDS-LiCl method from flag leaves of wheat plants grown under control (26 ℃/15 ℃) and HNT stress (26 ℃/23 ℃). Tx Control (**a)**, Tascosa Control **(b)**, Tx HNT stress **(c)** and Tascosa HNT stress **(d)**. X –axis units in nt (Nucleotides); Y –axis units in FU (Fluorescence Units). Tx- Tx86A5606, HNT – High night temperature.


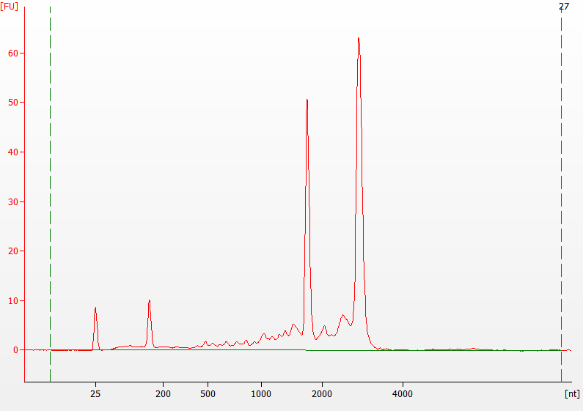


**20**

**10**

**0**

**30**

**40**

**50**

**[FU]**

**60**

**500**

**25**

**2000**

**200**

**1000**

**4000**

**[nt]**

**RIN: 8.30**

**18S**

**26S**

**c) Tascosa-germinated**

**seeds-control**


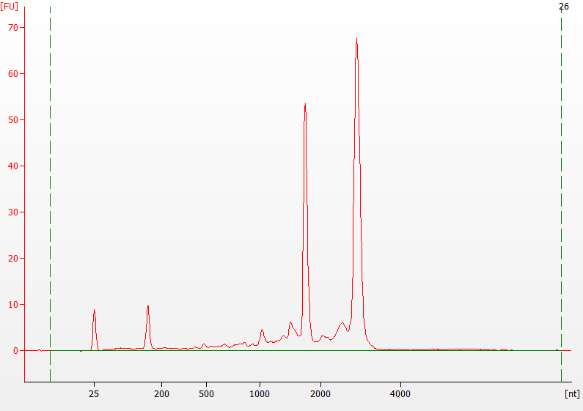


**20**

**10**

**0**

**30**

**40**

**50**

**[FU]**

**60**

**70**

**RIN: 8.70**

**18S**

**26S**

**b) Tx-germinated**

**seeds-cold stress**


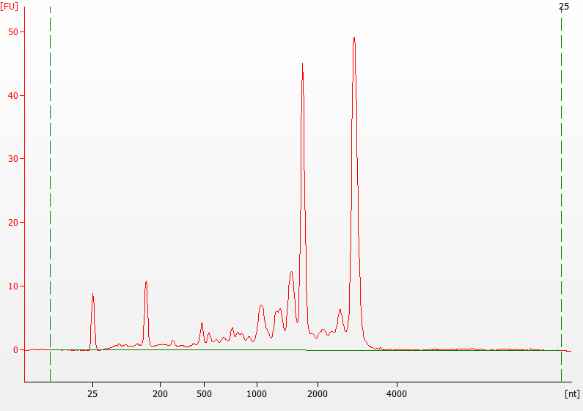


**20**

**10**

**0**

**30**

**40**

**50**

**[FU]**

**RIN: 7.10**

**18S**

**26S**

**a) Tx-germinated seeds-control**


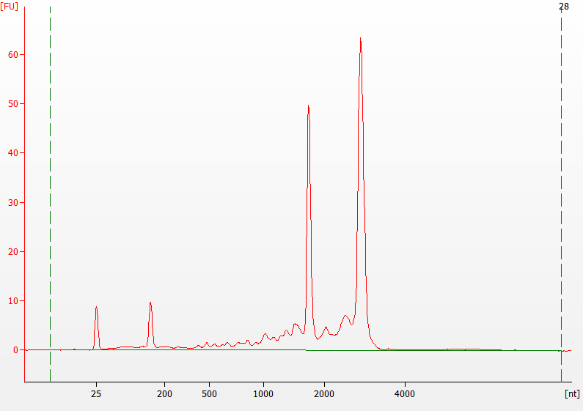


**20**

**10**

**0**

**30**

**40**

**50**

**[FU]**

**60**

**500**

**25**

**2000**

**200**

**1000**

**4000**

**[nt]**

**RIN: 8.40**

**18S**

**26S**

**d) Tascosa-germinated**

**seeds-cold stress**

**Supplementary Figure 11.** Electropherograms of total RNA isolated using modified SDS-LiCl method from germinated wheat seeds under control (30℃) and cold stress (15℃). Tx Control **(a)**, Tascosa Control **(b)**, Tx Cold stress **(c)** and Tascosa Cold stress **(d)**. X –axis units in nt (Nucleotides); Y –axis units in FU (Fluorescence Units). Tx- Tx86A5606.


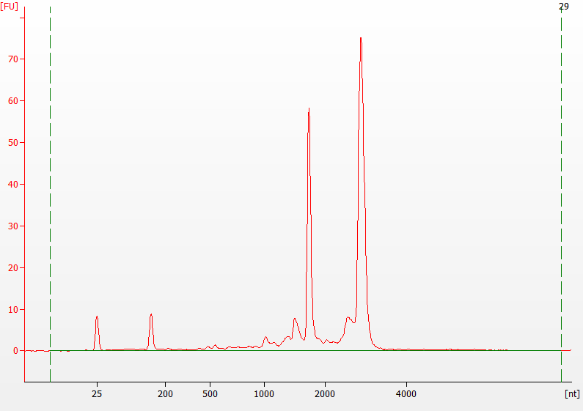


**20**

**10**

**0**

**30**

**40**

**50**

**[FU]**

**60**

**70**

**RIN: 8.90**

**18S**

**26S**

**a) Tx-root-control**


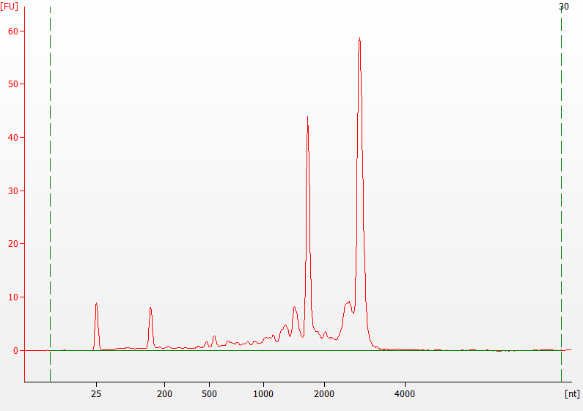


**20**

**10**

**0**

**30**

**40**

**50**

**[FU]**

**60**

**RIN: 8.10**

**18S**

**26S**

**b) Tx-root-freezing stress**


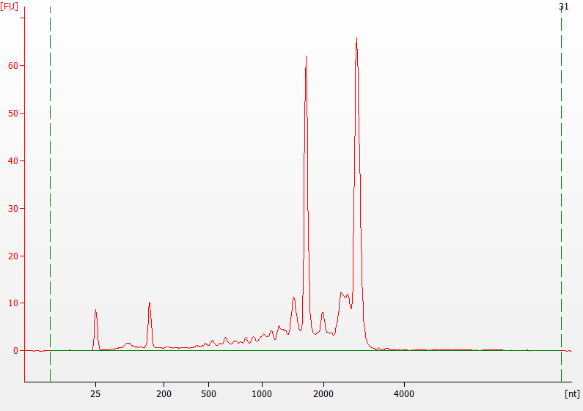


**20**

**10**

**0**

**30**

**40**

**50**

**[FU]**

**60**

**500**

**25**

**2000**

**200**

**1000**

**4000**

**[nt]**

**RIN: 7.60**

**18S**

**26S**

**c) Tascosa-root-control**


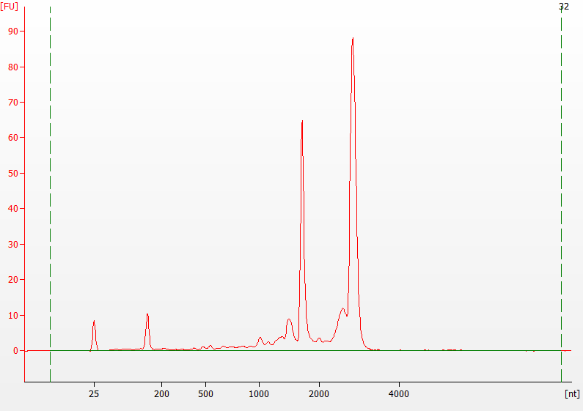


**20**

**10**

**0**

**30**

**40**

**50**

**[FU]**

**60**

**70**

**80**

**90**

**500**

**25**

**2000**

**200**

**1000**

**4000**

**[nt]**

**RIN: 8.80**

**18S**

**26S**

**d) Tascosa-root-freezing stress**

**Supplementary Figure 12.** Electropherograms of total RNA isolated using modified SDS-LiCl method from roots of wheat plants grown under control (25 ℃) and freezing stress (-4 ℃). Tx Control **(a)**, Tascosa Control **(b)**, Tx Freezing stress **(c)** and Tascosa Freezing stress **(d)**. X –axis units in nt (Nucleotides); Y –axis units in FU (Fluorescence Units). Tx- Tx86A5606.

**Supplementary Table 2.** Yield and quality of total RNA extracted using modified SDS-LiCl method from developing wheat seeds exposed to a range of increasing night temperatures (15-27 ℃) in controlled environment chambers. A common mean day temperature of 28 ℃ was maintained across all the night temperature treatments. RIN – RNA Integrity Numbers. FW – Fresh Weight.

| **Genotype**  **RNA sample** | **Nighttime temperature** | **RNA Yield (μg/100 mg FW)**  **range** | **A260/A280 ratio**  **range** | **A260/A230 ratio**  **range** | **RIN** |
| --- | --- | --- | --- | --- | --- |
| KS07077M-1 | 15 ℃ | 40.71 - 81.14 | 2.15 - 2.18 | 2.28 - 2.36 | 10.0 |
| KS07077M-1 | 23 ℃ | 13.71 - 78.66 | 2.13 - 2.18 | 2.22 - 2.37 | 9.60 |
| KS07077M-1 | 25 ℃ | 26.37 - 72.10 | 2.12 - 2.19 | 2.24 - 2.33 | 10.0 |
| KS07077M-1 | 27 ℃ | 17.72 - 34.83 | 2.08 - 2.18 | 1.74 - 2.29 | 7.90 |
|  |  |  |  |  |  |
| SY Monument | 15 ℃ | 12.36 - 40.12 | 2.11 - 2.19 | 1.67 - 2.39 | 9.80 |
| SY Monument | 23 ℃ | 14.84 - 60.35 | 2.08 - 2.19 | 2.21 - 2.40 | 10.0 |
| SY Monument | 25 ℃ | 15.48 - 91.71 | 2.09 - 2.16 | 2.14 - 2.30 | 10.0 |
| SY Monument | 27 ℃ | 16.20 - 29.09 | 2.08 - 2.17 | 2.21 - 2.38 | 9.50 |
|  |  |  |  |  |  |
| Larry | 15 ℃ | 53.05 - 75.26 | 2.14 - 2.19 | 2.27 - 2.32 | 10.0 |
| Larry | 23 ℃ | 37.35 - 76.45 | 2.16 - 2.20 | 2.21 - 2.24 | 7.80 |
| Larry | 25 ℃ | 16.80 - 84.05 | 2.12 - 2.16 | 2.17 - 2.29 | 9.60 |
| Larry | 27 ℃ | 64.04 - 92.25 | 2.08 - 2.17 | 2.21 - 2.30 | 8.30 |


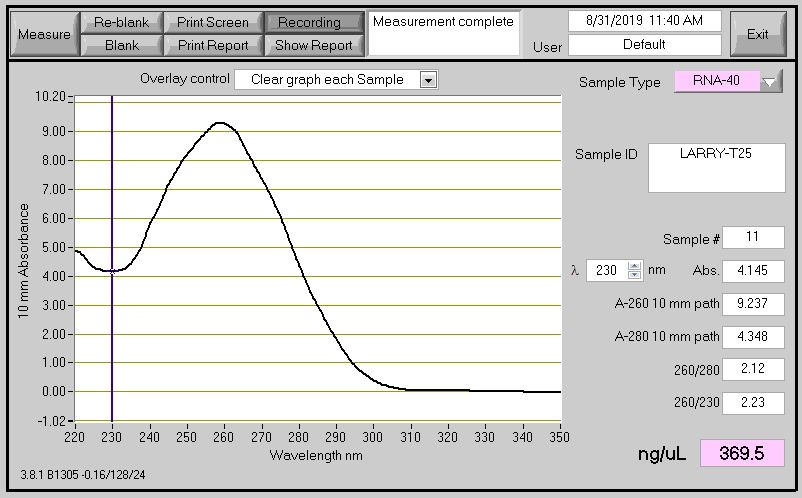


**c)**


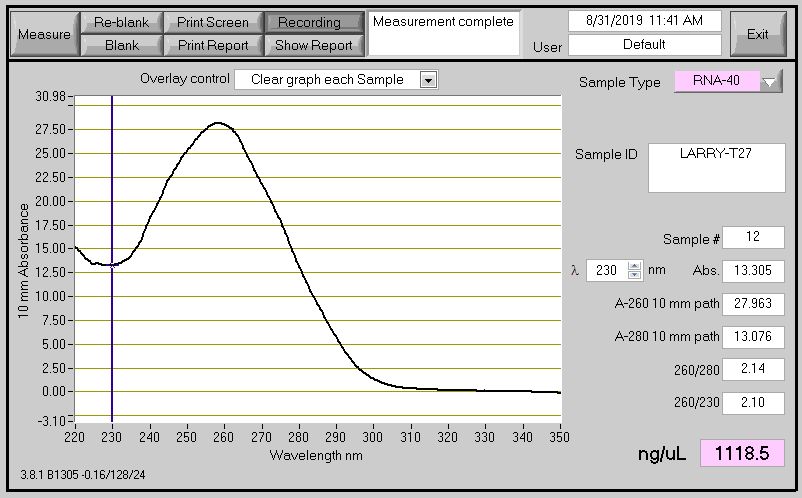


**d)**


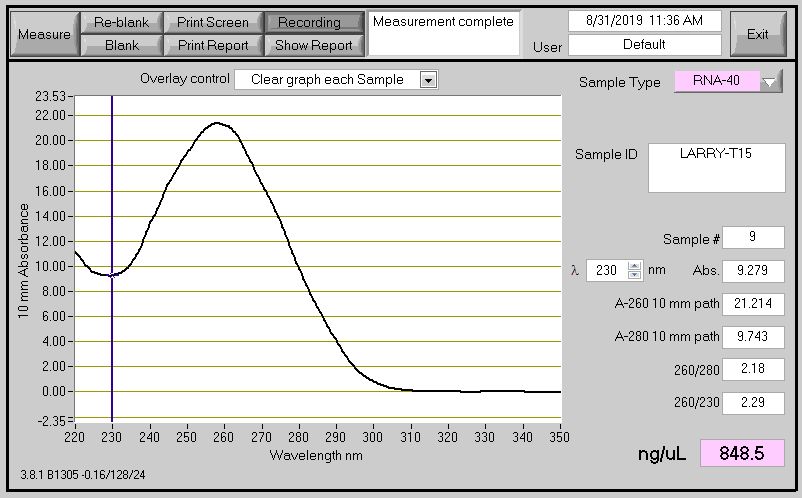


**a)**


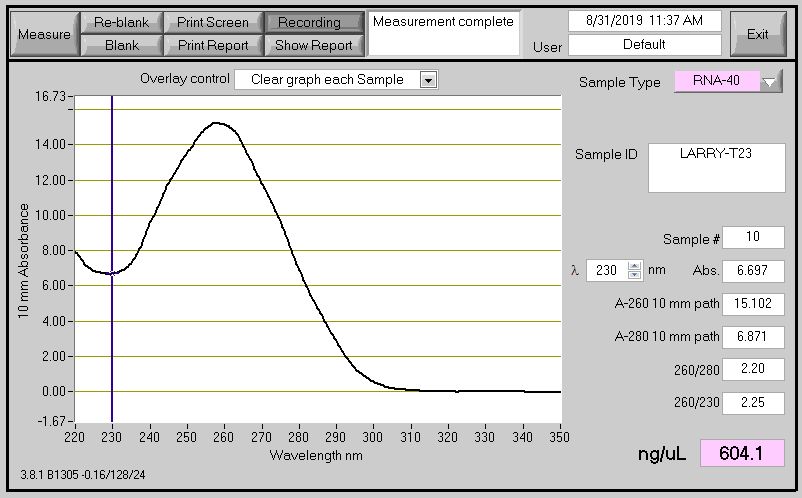


**b)**


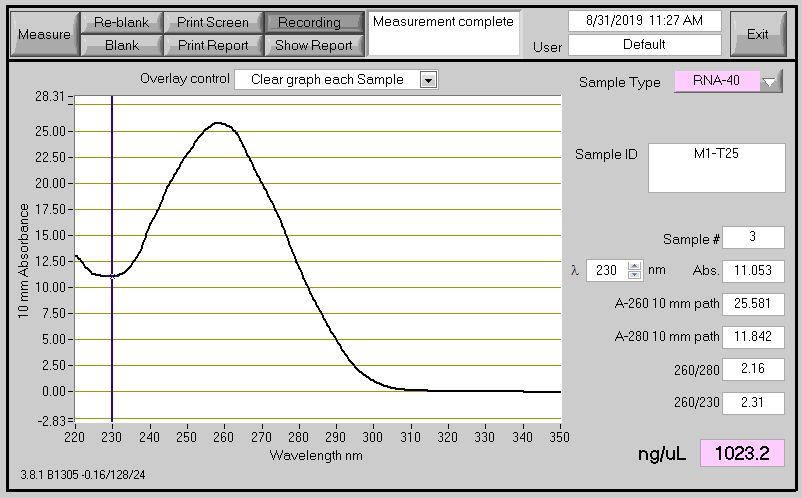


**g)**


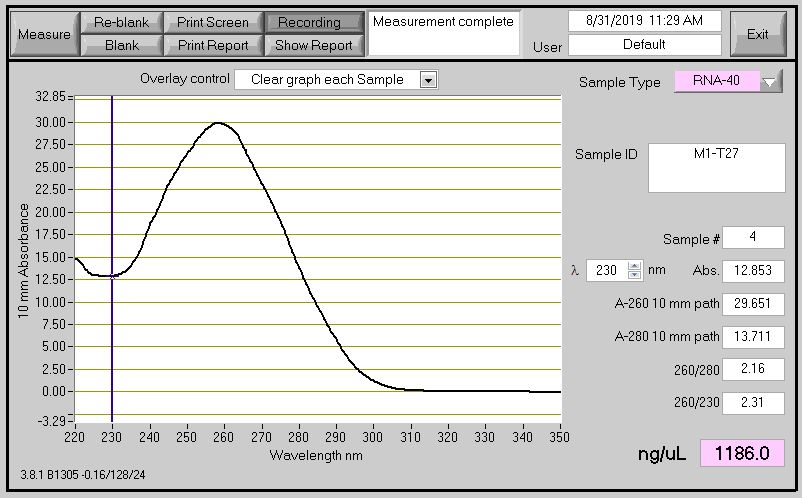


**h)**


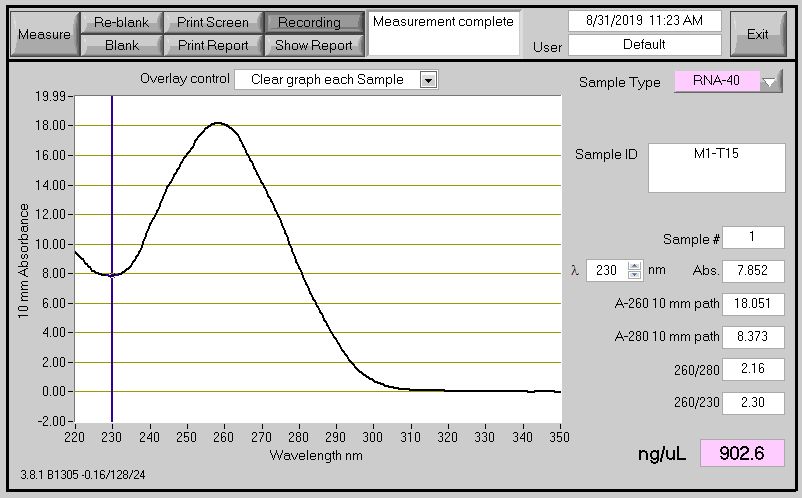


**e)**


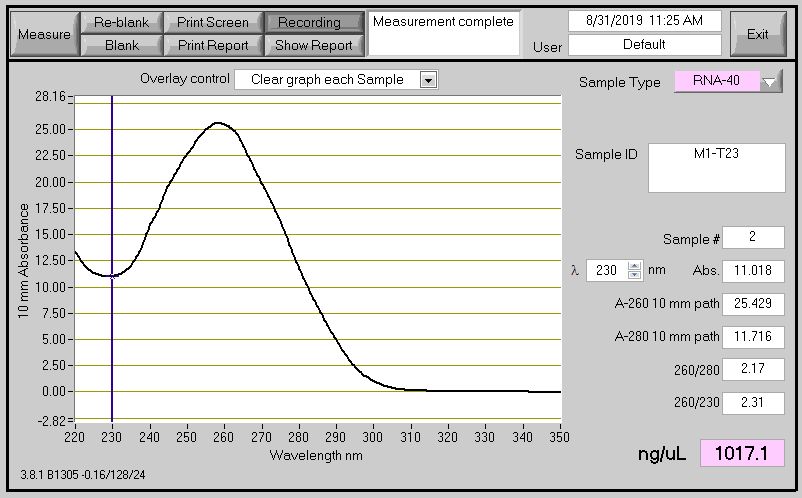


**f)**

**Supplementary Figure 13.** Absorbance spectrum of total RNA isolated using modified SDS-LiCl method from developing wheat seeds from Larry and KS07077M-1 exposed to different night temperature treatments, with a common day time temperature of 28 ^o^C. These absorbance spectra were obtained from RNA samples used for extensive grain starch metabolism gene expression profiling published (Impa et al.^20^). Larry 15 ℃ **(a)**, Larry 23 ℃ **(b)**, Larry 25 ℃ **(c)**, Larry 27 ℃ **(d)**, KS07077M-1 15 ℃ **(e)**, KS07077M-1 23 ℃ **(f)**, KS07077M-1 25 ℃ **(g)** and KS07077M-1 27 ℃ **(h)**.


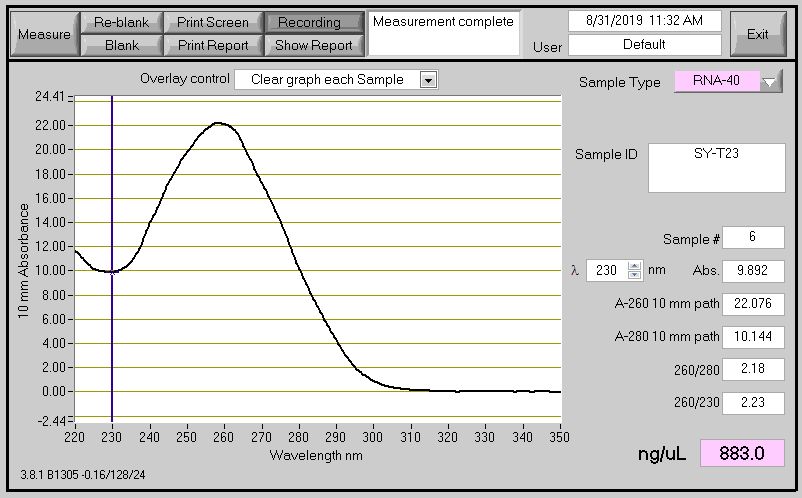


**b)**


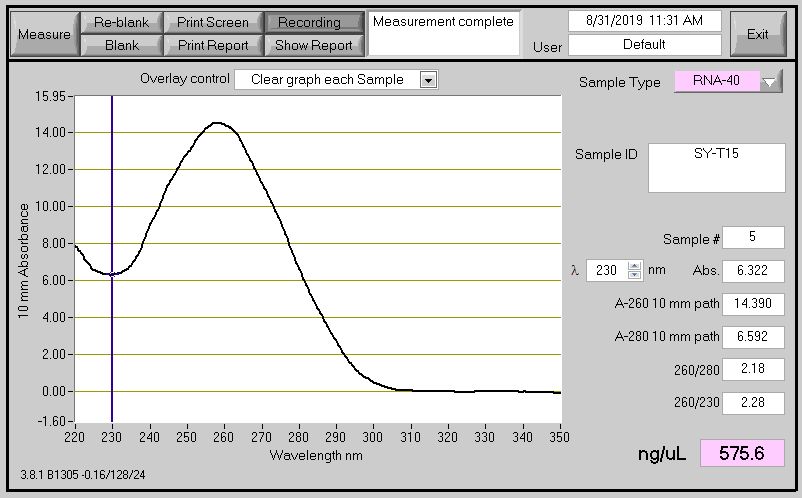


**a)**


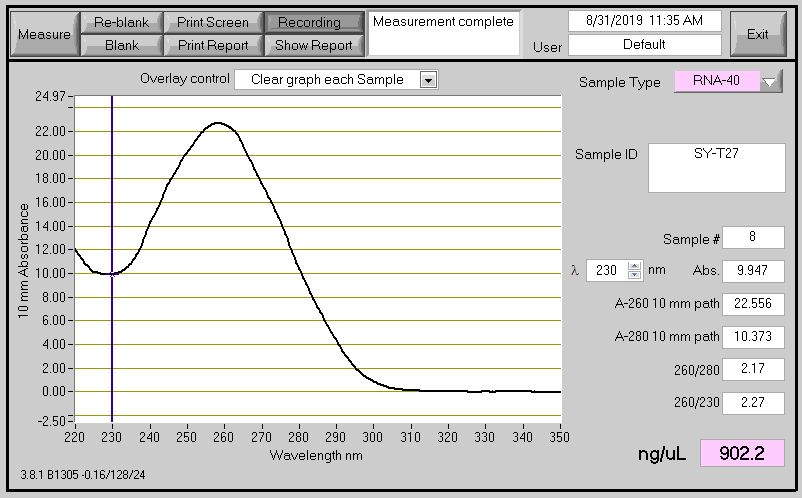


**d)**


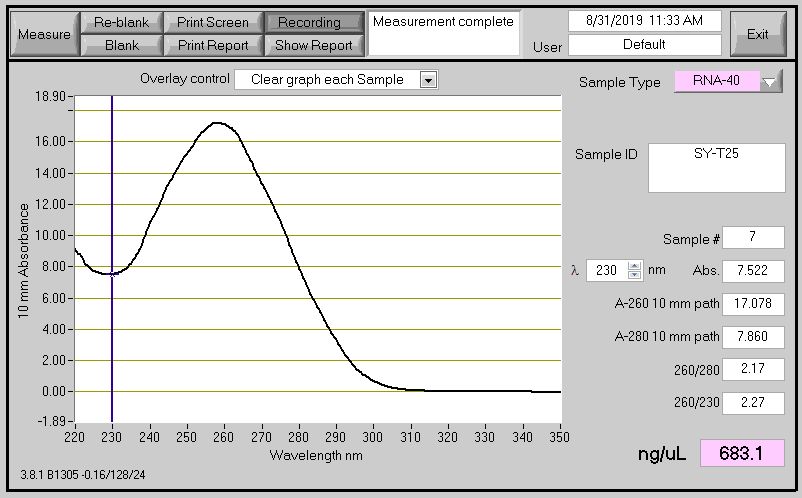


**c)**

**Supplementary Figure 14.** Absorbance spectrum of total RNA isolated using modified SDS-LiCl method from developing seeds of wheat genotype SY Monument exposed to different night temperature treatments, with a common day time temperature of 28 ^o^C. SY Monument 15 ℃ **(a)**, SY Monument 23 ℃ **(b)**, SY Monument 25 ℃ **(c)**, and SY Monument 27 ℃ **(d)**.


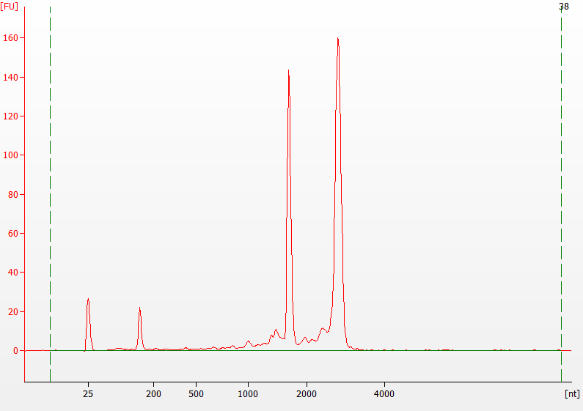


**20**

**0**

**[FU]**

**40**

**60**

**80**

**100**

**120**

**140**

**160**

**RIN: 9.60**

**18S**

**26S**

**b) KS07077M-1-**

**Developing grain-23 ℃**


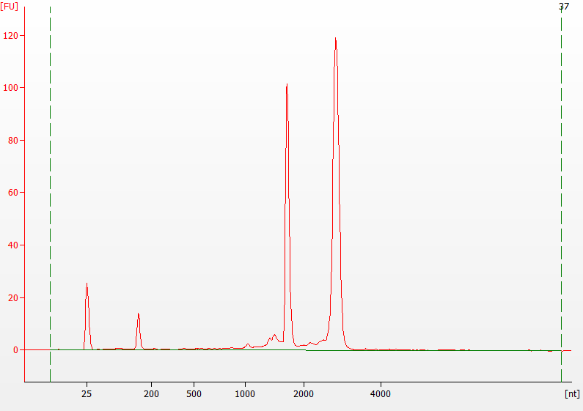


**20**

**0**

**[FU]**

**40**

**60**

**80**

**100**

**120**

**RIN: 10.0**

**18S**

**26S**

**a) KS07077M-1-**

**Developing grain-15 ℃**


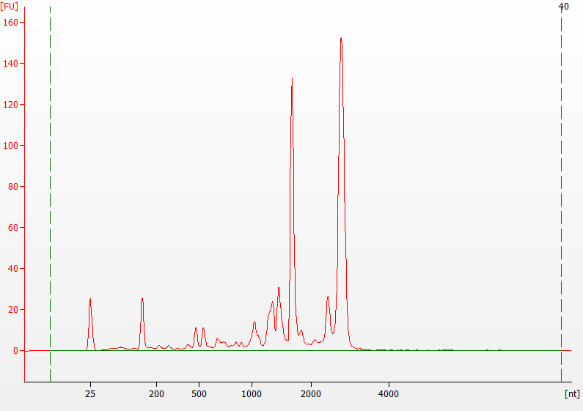


**20**

**0**

**[FU]**

**40**

**60**

**80**

**100**

**120**

**140**

**160**

**RIN: 7.90**

**18S**

**26S**

**500**

**25**

**2000**

**200**

**1000**

**4000**

**[nt]**

**d) KS07077M-1-**

**Developing grain-27 ℃**


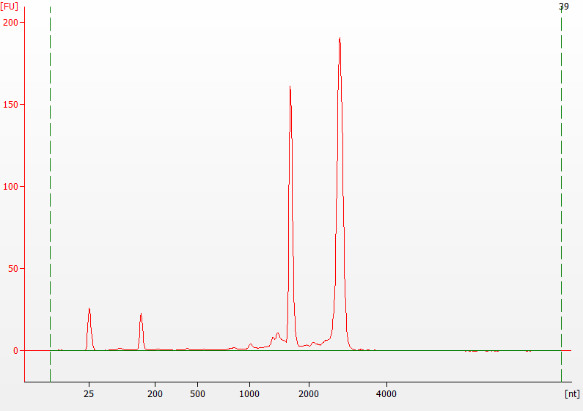


**0**

**[FU]**

**100**

**200**

**50**

**150**

**RIN: 10.0**

**18S**

**26S**

**500**

**25**

**2000**

**200**

**1000**

**4000**

**[nt]**

**c) KS07077M-1-**

**Developing grain-25 ℃**

**Supplementary Figure 15.** Electropherograms of total RNA isolated using modified SDS-LiCl method from developing seeds of wheat genotype KS07077M-1 exposed to various night temperature treatments, with a common day time temperature of 28 ^o^C. X –axis units in nt (Nucleotides); Y –axis units in FU (Fluorescence Units). KS07077M-1 15 ℃ **(a)**, KS07077M-1 23 ℃ **(b)**, KS07077M-1 25 ℃ **(c)** and KS07077M-1 27 ℃ **(d)**.


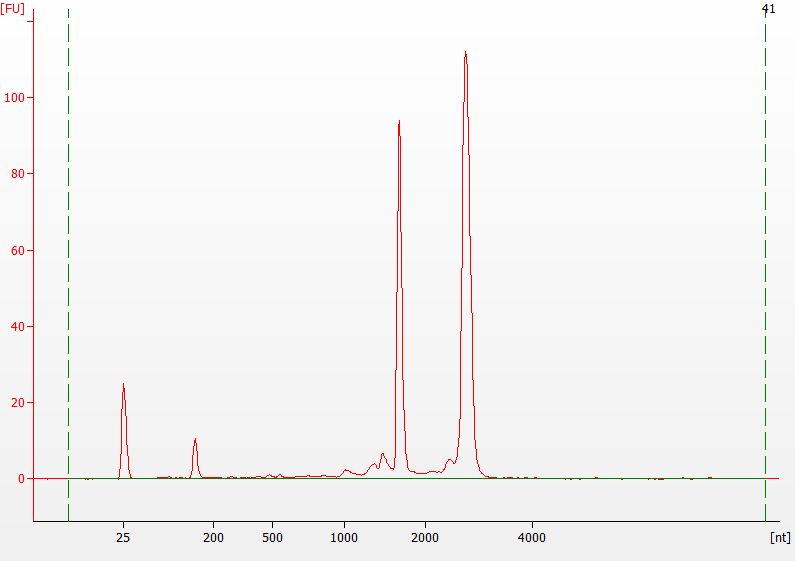


**20**

**0**

**[FU]**

**40**

**60**

**80**

**100**

**RIN: 9.80**

**18S**

**26S**

**a) SY Monument-**

**Developing grain-15 ℃**


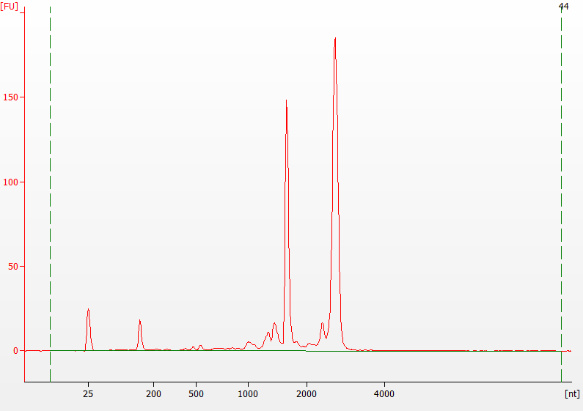


**0**

**100**

**50**

**150**

**500**

**25**

**2000**

**200**

**1000**

**4000**

**[nt]**

**[FU]**

**RIN: 9.50**

**18S**

**26S**

**d) SY Monument-**

**Developing grain-27 ℃**


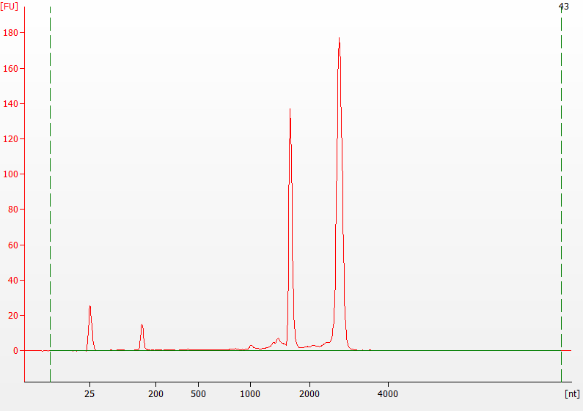


**20**

**0**

**[FU]**

**40**

**60**

**80**

**100**

**120**

**140**

**160**

**180**

**500**

**25**

**2000**

**200**

**1000**

**4000**

**[nt]**

**RIN: 10.0**

**18S**

**26S**

**c) SY Monument-**

**Developing grain-25 ℃**


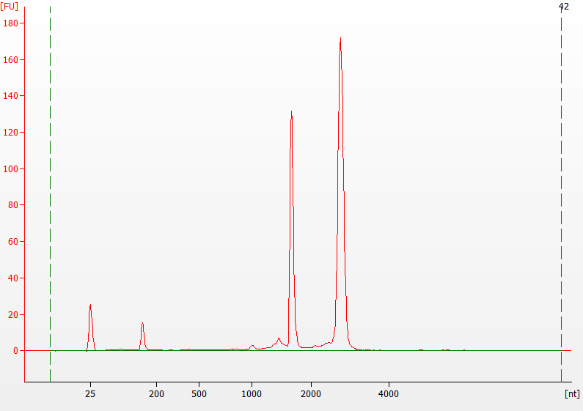


**20**

**0**

**[FU]**

**40**

**60**

**80**

**100**

**120**

**140**

**160**

**180**

**RIN: 10.0**

**18S**

**26S**

**b) SY Monument-**

**Developing grain-23 ℃**

**Supplementary Figure 16.** Electropherograms of total RNA isolated using modified SDS-LiCl method from developing seeds of wheat genotype SY Monument exposed to various night temperature treatments, with a common day time temperature of 28^o^C. X –axis units in nt (Nucleotides); Y –axis units in FU (Fluorescence Units). SY Monument 15 ℃ **(a)**, SY Monument 23 ℃ **(b)**, SY Monument 25 ℃ **(c)**, and SY Monument 27 ℃ **(d)**.


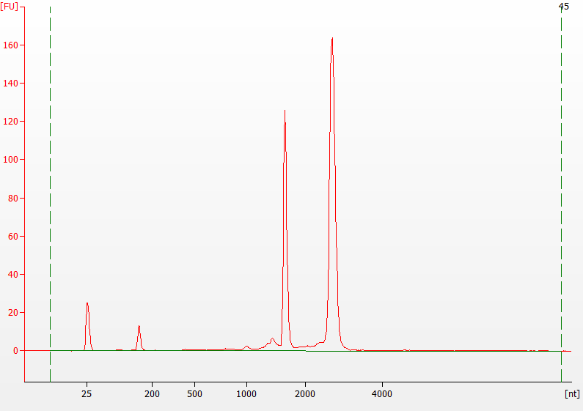


**20**

**0**

**[FU]**

**40**

**60**

**80**

**100**

**120**

**140**

**160**

**RIN: 10.0**

**18S**

**26S**

**a) Larry-**

**Developing grain-15 ℃**


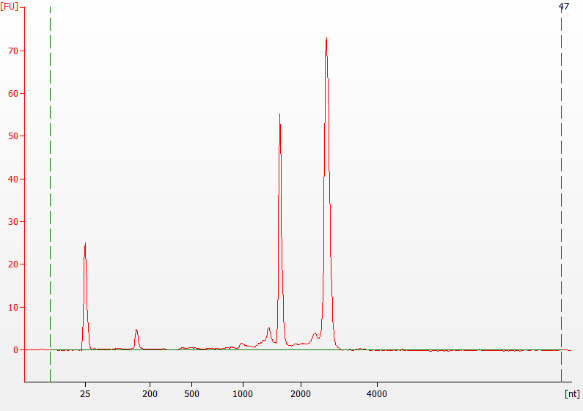


**20**

**10**

**0**

**30**

**40**

**50**

**[FU]**

**60**

**70**

**500**

**25**

**2000**

**200**

**1000**

**4000**

**[nt]**

**RIN: 9.60**

**18S**

**26S**

**c) Larry-**

**Developing grain-25 ℃**


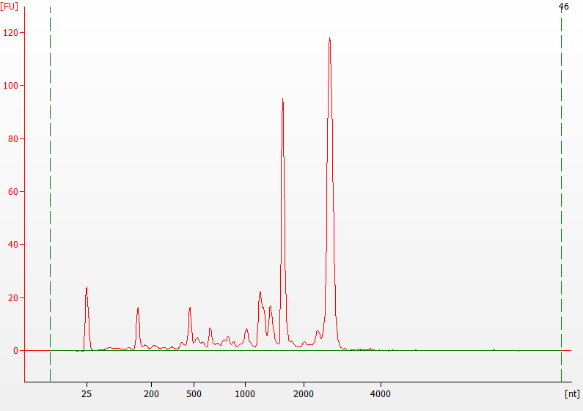


**20**

**0**

**[FU]**

**40**

**60**

**80**

**100**

**120**

**RIN: 7.80**

**18S**

**26S**

**b) Larry-**

**Developing grain-23 ℃**


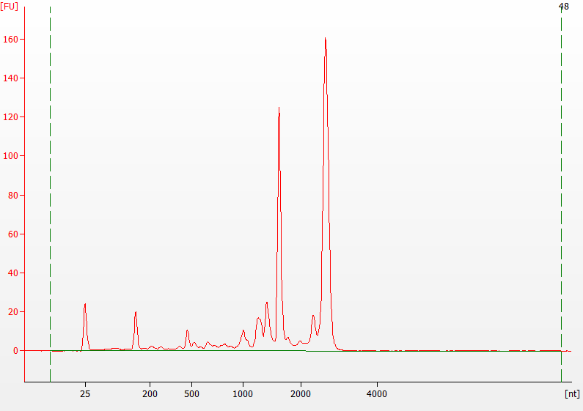


**20**

**0**

**[FU]**

**40**

**60**

**80**

**100**

**120**

**140**

**160**

**500**

**25**

**2000**

**200**

**1000**

**4000**

**[nt]**

**RIN: 8.30**

**18S**

**26S**

**d) Larry-**

**Developing grain-27 ℃**

**Supplementary Figure 17.** Electropherograms of total RNA isolated using modified SDS-LiCl method from developing seeds of wheat genotype Larry exposed to different night temperature treatments, with a common day-time temperature of 28 ^o^C. X –axis units in nt (Nucleotides); Y –axis units in FU (Fluorescence Units). Larry 15 ℃ **(a)**, Larry 23 ℃ **(b)**, Larry 25 ℃ **(c)**, Larry 27 ℃ **(d)**.

**
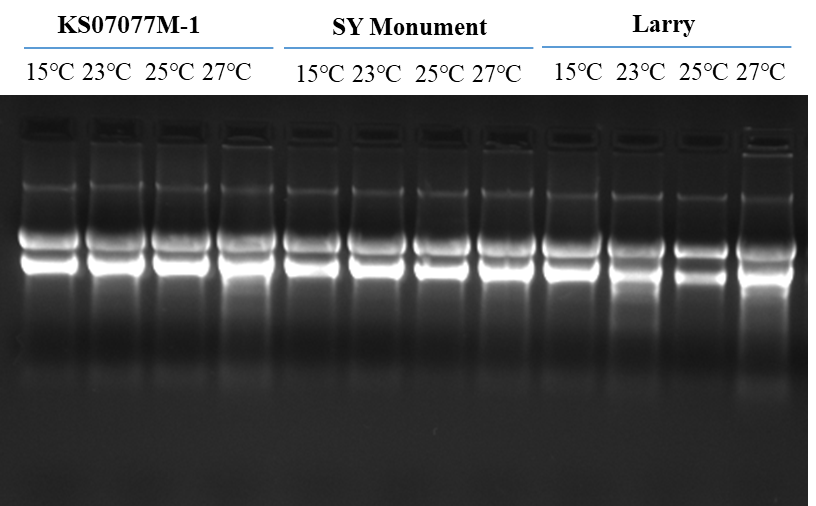
**

**Supplementary Figure 18.** Agarose gel electrophoresis of total RNA isolated using modified SDS-LiCl method from developing seeds of wheat plants exposed to 15 ℃, 23 ℃, 25 ℃ and 27 ℃ night temperature treatments, with a common day time temperature of 28 ^o^C. The total RNA was extracted from wheat seeds collected from KS07077M-1, SY Monument and Larry at 14 days of HNT stress imposition during grain filling.


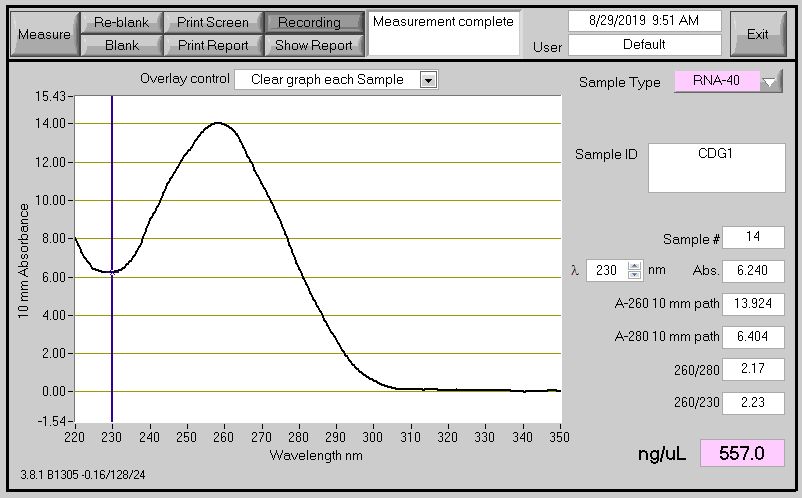

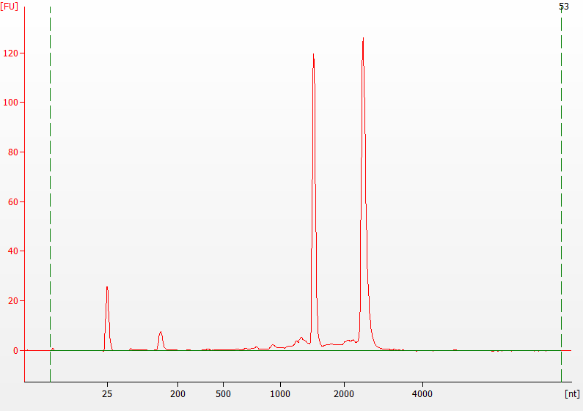


**20**

**0**

**[FU]**

**40**

**60**

**80**

**100**

**120**

**[nt]**

**500**

**25**

**2000**

**200**

**1000**

**4000**

**RIN: 8.10**

**18S**

**26S**

**a)**


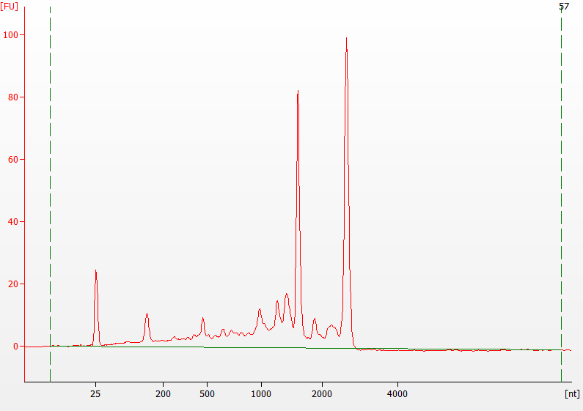


**20**

**0**

**[FU]**

**40**

**60**

**80**

**100**

**500**

**25**

**2000**

**200**

**1000**

**4000**

**[nt]**

**RIN: 7.00**

**18S**

**26S**

**b)**


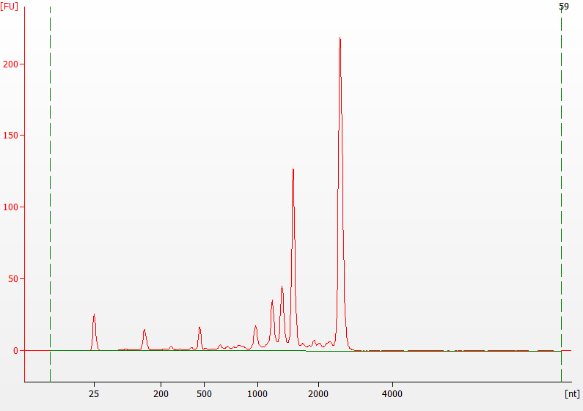


**0**

**100**

**200**

**50**

**150**

**500**

**25**

**2000**

**200**

**1000**

**4000**

**[nt]**

**[FU]**

**RIN: 8.20**

**18S**

**26S**

**c)**


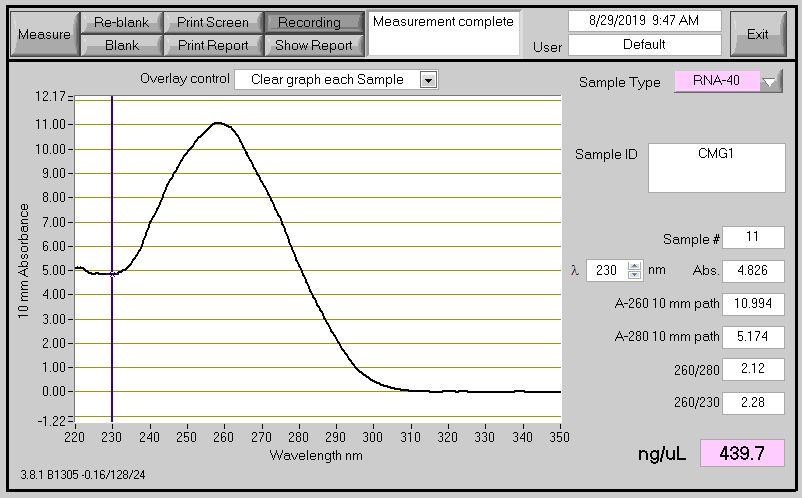


**e)**


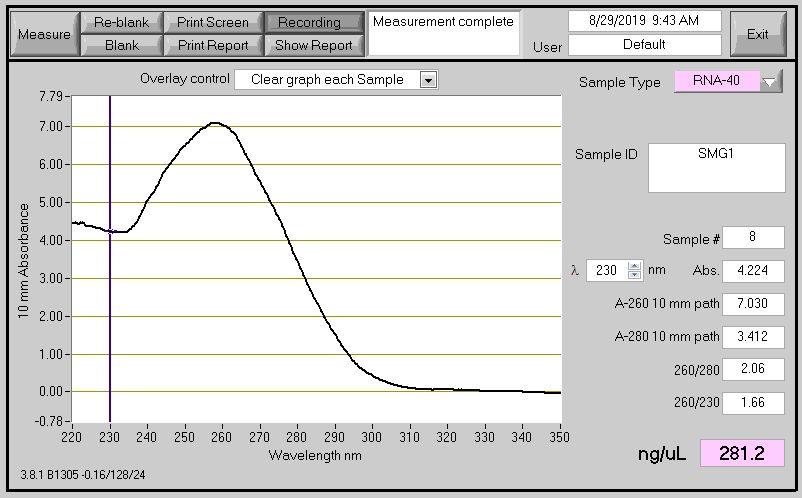


**f)**

**d)**

**Supplementary Figure 19.** Bioanalyzer and nano-spectrophotometric analysis of the total RNA isolated using the modified SDS-LiCl method from developing sorghum seeds, and mature and developing maize seeds from plants grown under field conditions. Electropherograms of total RNA of developing seeds of sorghum **(a)**, developing seeds of maize (**b**), and mature seeds of maize (**c**). X –axis units in nt (Nucleotides); Y –axis units in FU (Fluorescence Units). Absorbance spectrum of total RNA of developing seeds of sorghum (**d**), developing seeds of maize (**e**), and mature seeds of maize (**f**).


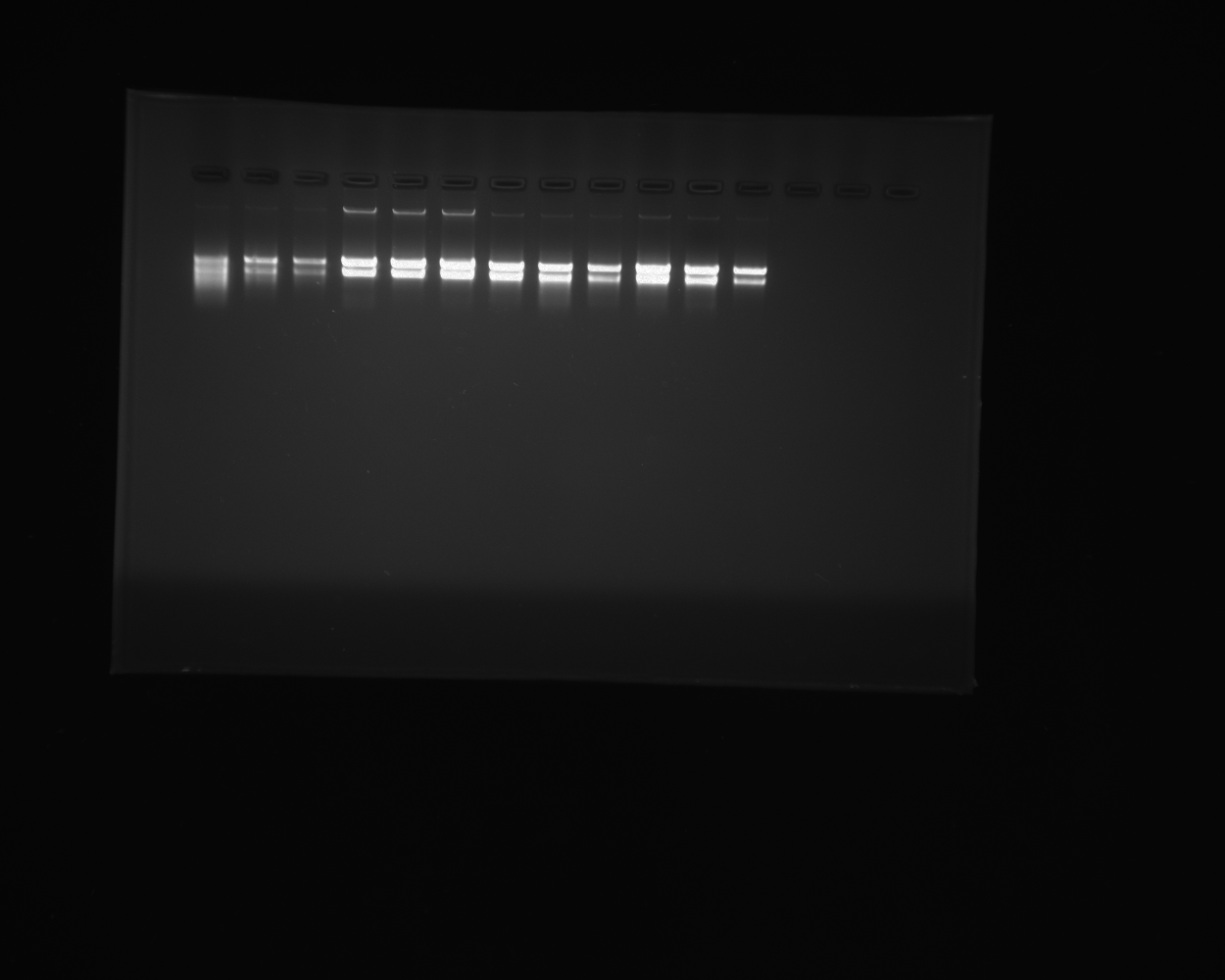


**Developing Sorghum**

**seeds**

**Developing Corn**

**seeds**

**Mature Corn**

**seeds**

**Supplementary Figure 20.** Agarose gel electrophoresis of the total RNA extracted using the modified SDS-LiCl method from developing seeds of sorghum, developing and mature seeds of maize grown under field conditions.


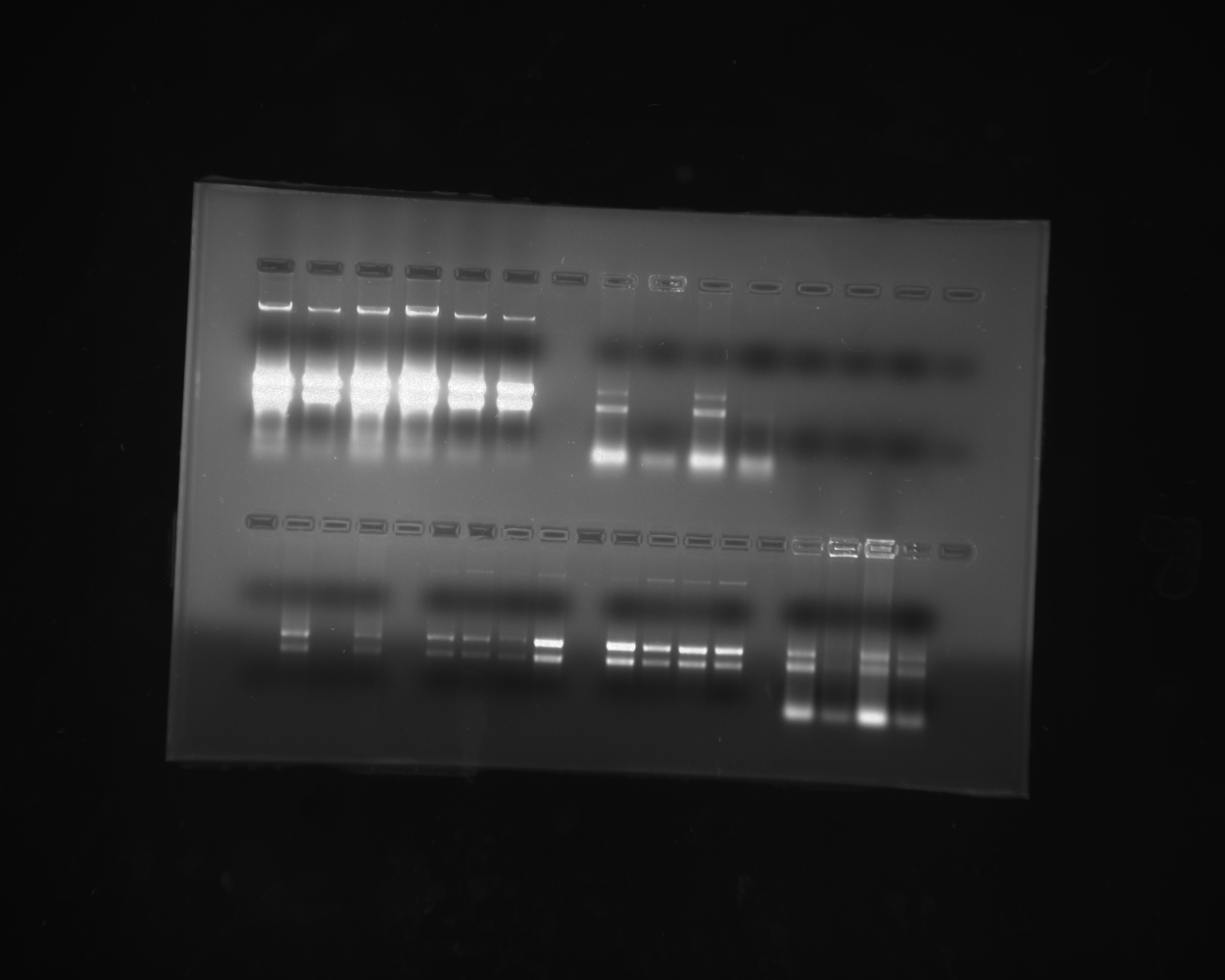


**a) Ambion TRIZOL**

**b) RNeasyPlant Mini Kit-Qiagen**

**c) Furtado, 2014**

**d) CTAB-LiCl**

**e) Modified SDS-LiCl**

**1 2 3 4**

**1 2 3 4**

**1 2 3 4**

**1 2 3 4**

**1 2 3 4**

**Supplementary source figure 1:** In this original source RNA gel image, the RNA extracted from mature wheat seeds using a) Ambion TRIZOL, b) RNeasy Plant Mini Kit (Qiagen), c) Furtado, 2014 and d) CTAB-LiCl methods were documented and represented in the blue boxes for the selected genotypes were cropped and used for the figure 2a-d. The documentation of gel picture at high contrast to detect the RNA (extracted using 5 different RNA extraction protocols) bands with low intensity on the gel resolved by the agarose gel electrophoresis. Total RNA was extracted from mature wheat seeds collected from plants grown under ambient and post-flowering high night temperature (HNT) stress conditions in the field. The five extraction methods are Ambion TRIZOL (a), RNeasy Plant Mini Kit (Qiagen) (b), Furtado, 2014^6^ method (c), CTAB-LiCl method (d), and modified SDS-LiCl method (e). The total RNA from wheat seeds of four different genotypes are numerically labeled from 1 to 4. 1: Tx Control, 2: Tx HNT stress, 3: Tascosa Control and 4: Tascosa HNT stress. Tx- Tx86A5606.


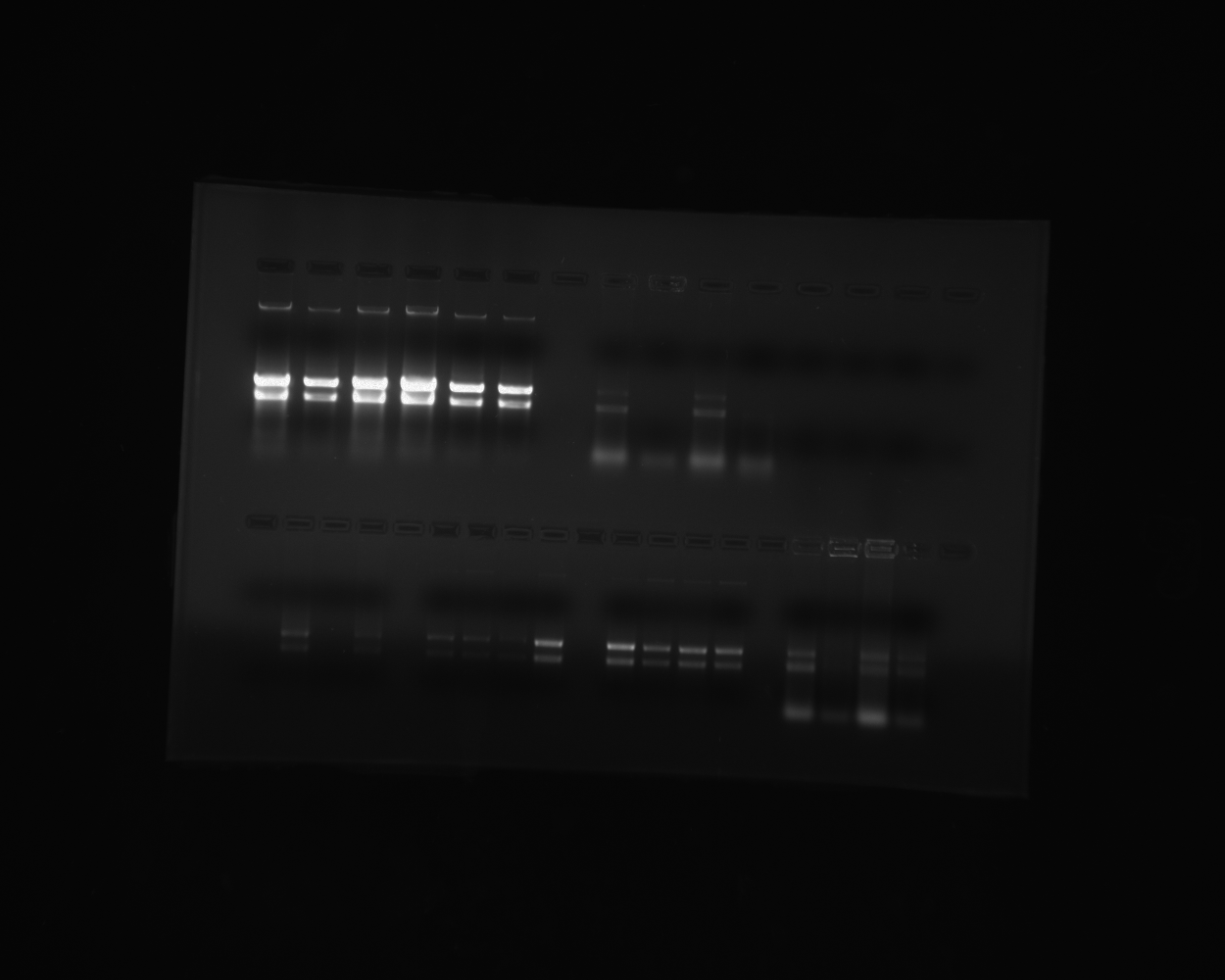


**a) Ambion TRIZOL**

**b) RNeasy Plant Mini Kit-Qiagen**

**c) Furtado, 2014**

**d) CTAB-LiCl**

**e) Modified SDS-LiCl**

**Supplementary source figure 2:** In this figure, the only e) modified SDS-LiCl RNA gel image showed in the blue box were cropped and used for the figure 2e. The gel picture shows the agarose gel documented at low contrast to detect the RNA (isolated using 5 different RNA extraction methods) bands with high intensity on the gel resolved by the agarose gel electrophoresis. Total RNA was extracted from mature wheat seeds collected from plants grown under ambient and post-flowering high night temperature (HNT) stress conditions in field. The five extraction methods are Ambion TRIZOL (a), RNeasy Plant Mini Kit (Qiagen) (b), Furtado, 2014 method^6^ (c), CTAB-LiCl method (d), and modified SDS-LiCl method (e). The total RNA from wheat seeds of four different genotypes are numerically labeled from 1 to 4. 1: Tx Control, 2: Tx HNT stress, 3: Tascosa Control and 4: Tascosa HNT stress. Tx- Tx86A5606.


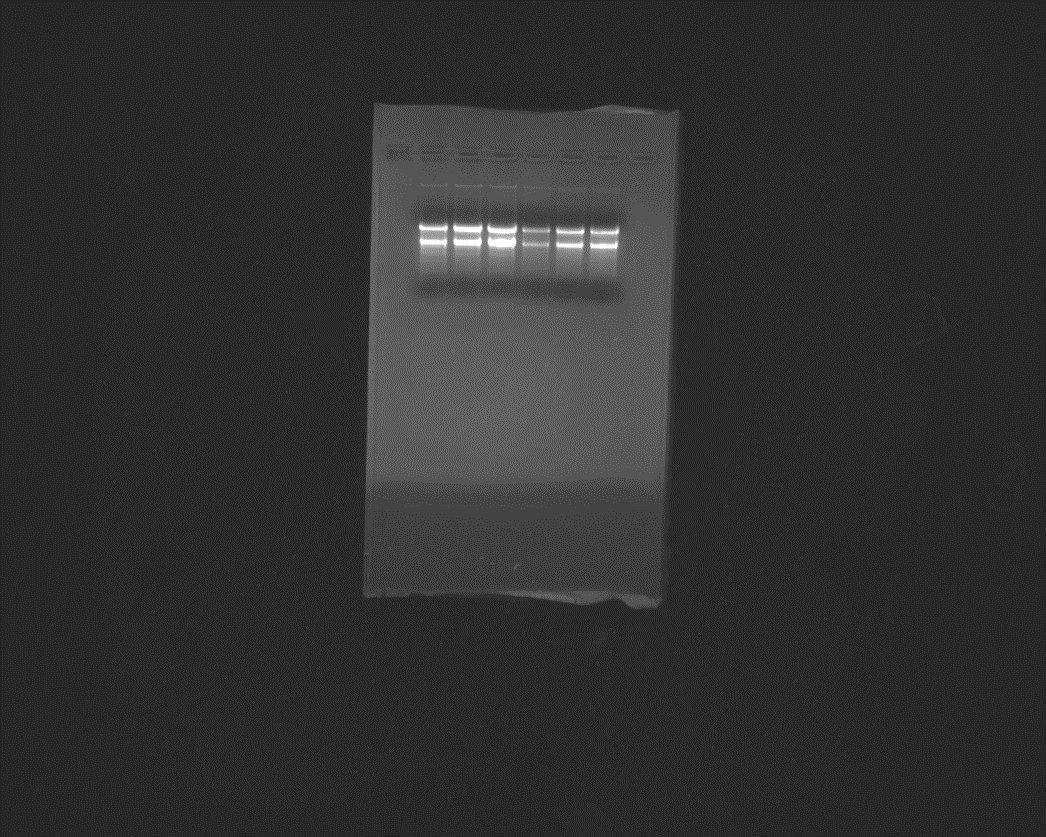

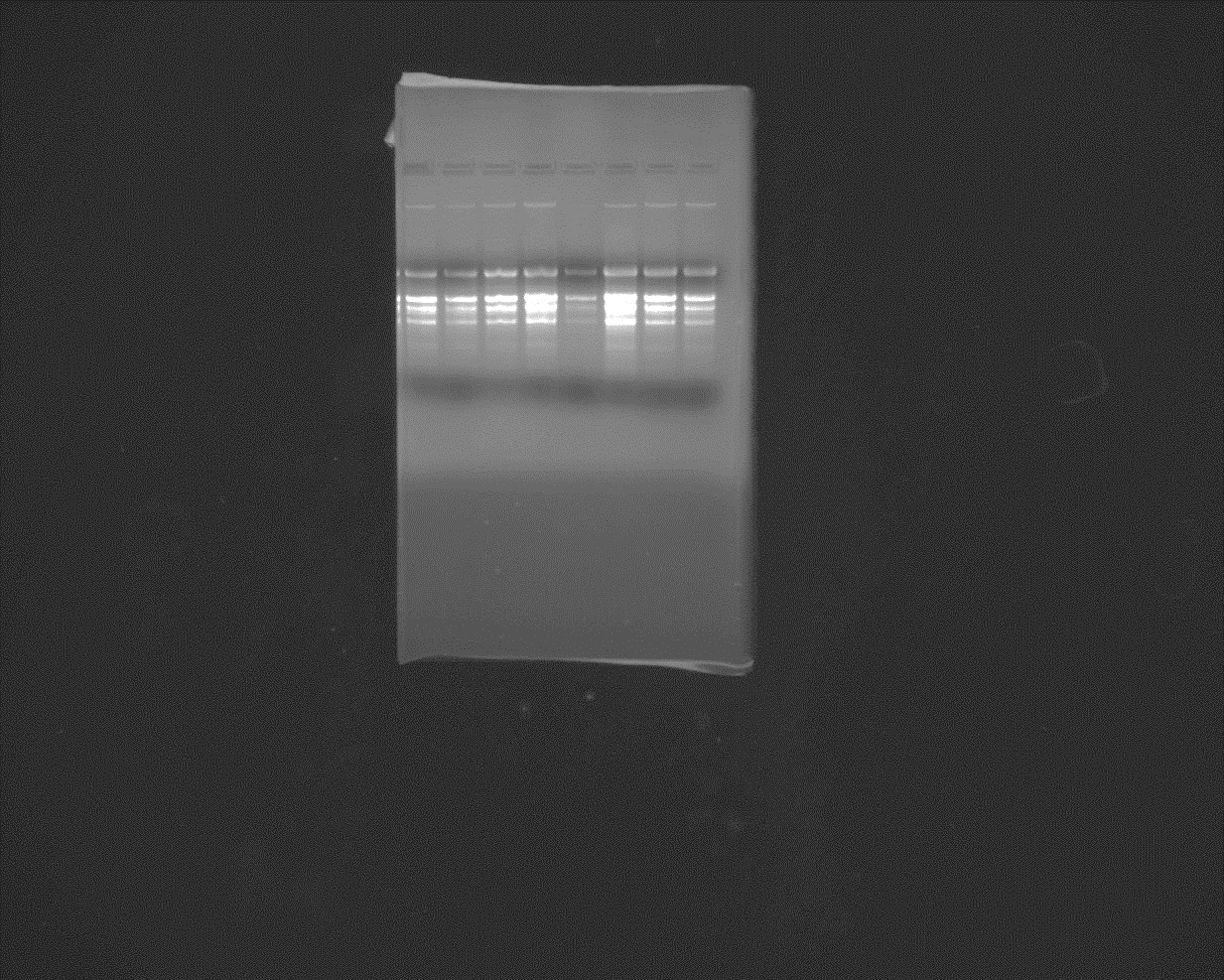

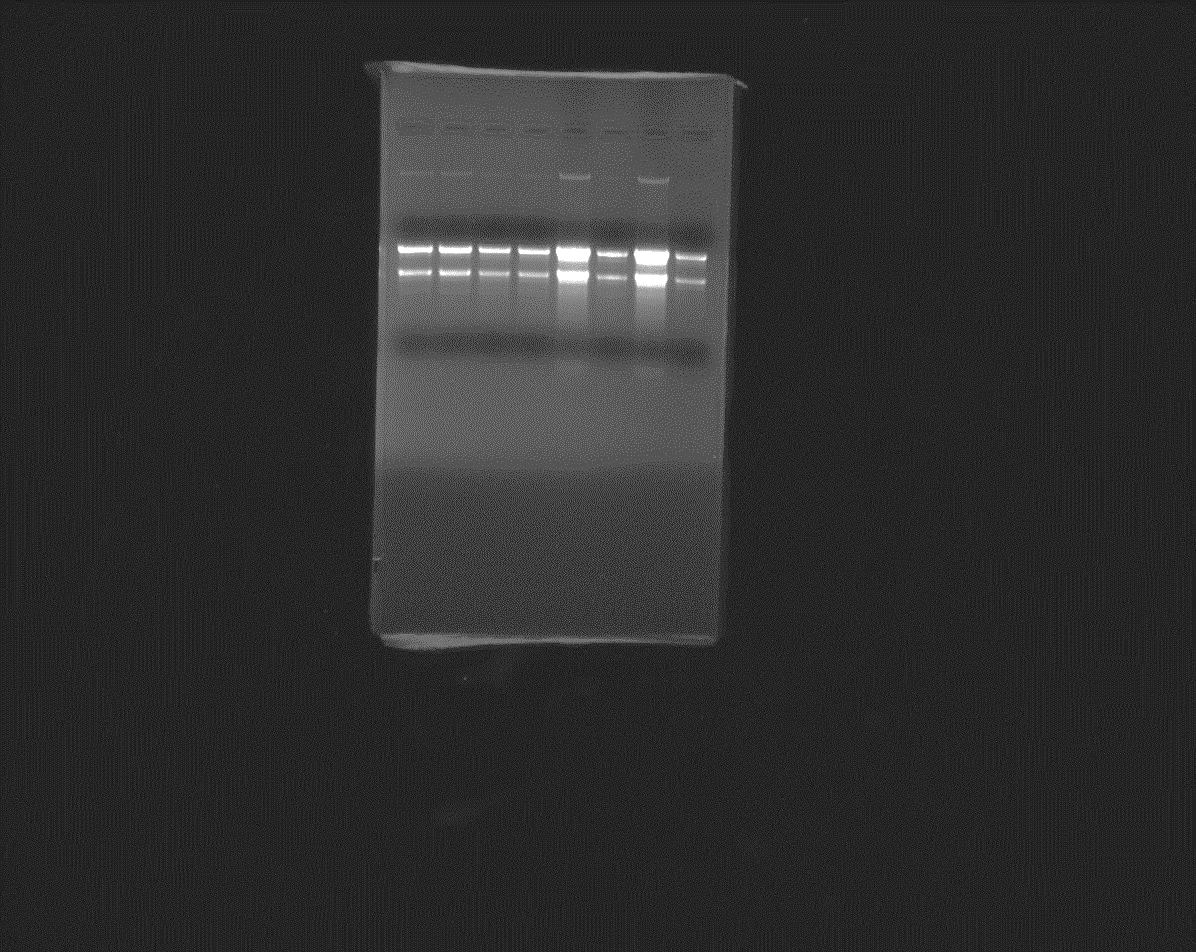


**a) Germinated seeds**

**-Cold Stress**

**b) Leaf - HNT Stress**

**c) Root - Cold Stress**

**1 2 3 4 5 6 7 8**

**1 2 3 4 5 6 7 8**

**1 2 3 4 5 6**

**Supplementary source figure 3:** The original source gel pictures documented at certain exposure to detect the RNA on the gel resolved by the agarose electrophoresis. Original pictures shows the agarose gel electrophoresis of total RNA extracted using the modified SDS-LiCl extraction method from germinated wheat seeds (a), flag leaf (b) and root (c) tissues exposed to different abiotic stresses. RNA extracted from two genotypes of wheat seedlings germinated under control (30 ℃) and cold stress conditions (15 ℃) (a), Lane 1: Tx- control, Lane 2-3: Tx- cold stress, Lane 4: Tascosa Control, Lane: 5-6 Tascosa Cold stress. RNA isolated from the flag leaves of two wheat genotypes grown under control (26 ℃/15 ℃) and HNT stress (26 ℃/23 ℃) (b), Lane 1-2: Tx Control, Lane 3-4: Tascosa Control, Lane 5-6: Tx HNT stress, Lane 7-8: Tascosa- HNT stress. RNA extracted from the roots of wheat genotypes grown under control (25 ℃) and freezing stress (-4 ℃) (c), Lane 1-2: Tx Control, Lane 3-4: Tx Freezing stress, Lane 5-6: Tascosa Control, Lane 7-8: Tascosa Freezing stress. Tx- Tx86A5606.
